# Polo-like kinase 1–inhibitor co-complex structures via the surface-entropy reduction approach and a DARPin-assisted approach

**DOI:** 10.1107/S2059798325009325

**Published:** 2025-11-17

**Authors:** Uwe Eberspaecher, Arndt A. Schmitz, Gerhard Siemeister, Ulf Bömer, Tiago M. Bandeiras, Pedro M. Matias, Volker K. Schulze, Roman C. Hillig

**Affiliations:** a Research and Development, Pharmaceuticals, Bayer AG, Muellerstrasse 178, 13353Berlin, Germany; b Nuvisan ICB GmbH, Muellerstrasse 178, 13353Berlin, Germany; c iBET, Instituto de Biologia Experimental e Tecnológica, Apartado 12, 2781-901Oeiras, Portugal; National Hellenic Research Foundation, Greece

**Keywords:** surface-entropy reduction, SER, kinase, crystallization, PLK1 small-molecule inhibitor

## Abstract

Polo-like kinase 1 (PLK1) is a major regulator of cell division and has been pursued as a drug target for cancer therapy for a long time. Crystallization of the kinase domain has proven to be exceptionally challenging. Previously, we published a crystallization approach using a PLK1-specific designed ankyrin-repeat protein (DARPin) as a crystallization facilitator. Here, we report an alternative route: crystallization was successful after the introduction of a double mutation which reduced surface entropy and enabled the formation of a new crystal contact. This new PLK1 crystallization system was used to determine the first co-complex crystal structure of the Bayer thiazolidinone lead series, as well as crystal structures with representatives of two competitor inhibitor series. The molecular binding modes of these three inhibitors are analysed and discussed, and the surface-entropy reduction approach is compared with the surface modifications employed by us and others to enable the crystallization of PLK1.

## Abstract 2

A surface-entropy reduction approach for human Polo-like kinase 1 (PLK1) enabled a reproducible and robust crystallization system for this cancer drug target. Co-crystal structures with representatives of three different small-molecule inhibitor classes were elucidated. For two of them, co-crystal structures with PLK1 were also determined via a previously published crystallization approach using a designed ankyrin-repeat protein (DARPin), and the two approaches are compared.

## 1. Introduction

**Figure 1:**
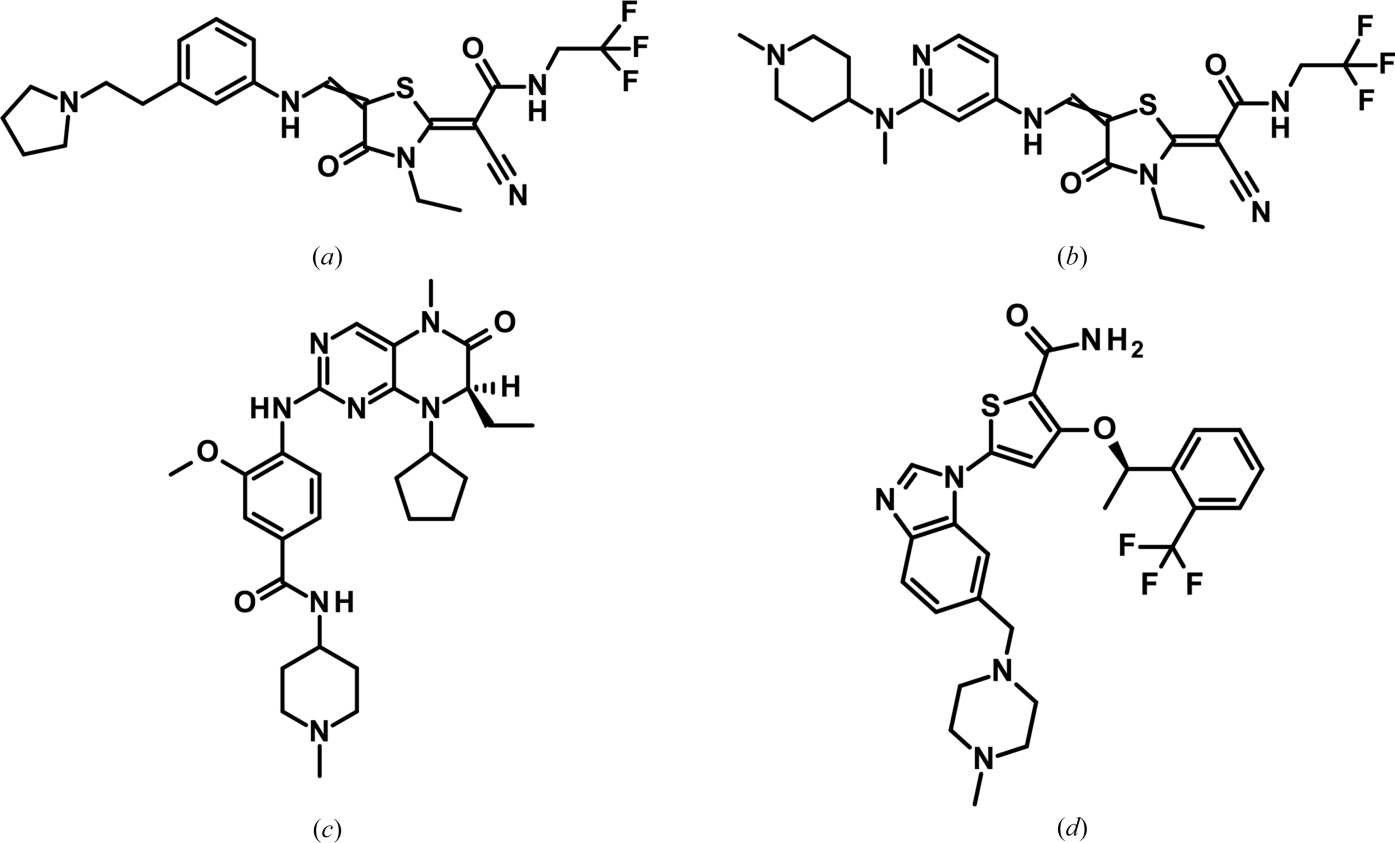
Chemical structures of PLK1 inhibitors discussed in this study. (*a*) ZK-Thiazolidinone (TAL; Santamaria *et al.*, 2007 ▸); (*b*) the closely related thiazolidinone inhibitor compound 1; (*c*) BI 2536 (Lénárt *et al.*, 2007 ▸; Steegmaier *et al.*, 2007 ▸; Kothe, Kohls, Low, Coli, Rennie *et al.*, 2007 ▸); (*d*) inhibitor GSK461364 (Olmos *et al.*, 2011 ▸).

Polo-like kinase 1 (PLK1) is a serine/threonine kinase which regulates the progression of cells through mitosis (Barr *et al.*, 2004 ▸). It is highly expressed in a broad range of human tumours, and this is associated with a poor prognosis in several types of cancer (Eckerdt *et al.*, 2005 ▸; Takai *et al.*, 2005 ▸). This association with tumorigenesis suggests PLK1 as an attractive target for cancer therapy (Liu, 2015 ▸). In a high-throughput screening approach, we previously identified a thiazolidinone lead series represented by ZK-Thiazolidinone (TAL; Fig. 1 ▸) as potent inhibitors of PLK1 (Santamaria *et al.*, 2007 ▸). TAL was shown to inhibit human PLK1 in a biochemical assay with an IC_50_ of 19 ± 12 n*M* and showed high kinase selectivity, only inhibiting the closely related kinases PLK2 and PLK3 in a panel of 93 tested serine/threonine and tyrosine kinases (Santamaria *et al.*, 2007 ▸).

Efforts to support our PLK1 drug-discovery program with crystal structure information were initially hampered since wild-type PLK1 resisted crystallization efforts. Reflecting these challenges, we initiated parallel activities to enable a crystallographic system. In a first approach, which we have reported previously (Bandeiras *et al.*, 2008 ▸), a PLK1-selective stabilizing designed ankyrin-repeat protein (DARPin) was generated and, upon complex formation with PLK1, enabled crystallization and structure determination of the wild-type PLK1 kinase domain. In a second, parallel approach, which we present in this contribution, a surface-entropy reduction (SER) mutant of PLK1 also yielded a functional crystallo­graphic system. The SER approach postulates that clusters of surface-exposed residues with side chains with many rotatable bonds, such as lysine, glutamate, glutamine and arginine, lose too much entropy upon crystal contact formation and therefore hinder crystallization (Derewenda, 2004 ▸, 2010 ▸; Derewenda & Vekilov, 2006 ▸; Winn *et al.*, 2011 ▸; Cooper *et al.*, 2007 ▸). Crystallization can be facilitated when such surface clusters are mutated to residues with smaller, less entropically active side chains. We have recently reported two case studies in which an SER approach enabled crystallization of the protein kinases Aurora-C and BUB1 (Schaefer *et al.*, 2024 ▸). In the kinase field, this has also been described, for example, for the kinase domain of the insulin-like growth factor receptor IGFR1 (Munshi *et al.*, 2003 ▸) and the human vaccine-related kinase 1 (VRK1; Couñago *et al.*, 2017 ▸). Here, we report a new crystal form of PLK1 that was obtained after the mutation of two adjacent surface lysine residues (Lys225 and Lys226). In order to validate both the DARPin and the SER approach, we selected two small-molecule inhibitors as tool compounds: the thiazolidinone compound 1 ((2*Z*)-2-cyano-2-{3-ethyl-5-[({2-[methyl(1-methylpiperidin-4-yl)amino]pyridin-4-yl}amino)methylidene]-4-oxo-1,3-thiazolidin-2-ylidene}-*N*-(2,2,2-trifluoroethyl)acetamide) from Bayer’s TAL series (Santamaria *et al.*, 2007 ▸) and BI 2536 from the Boehringer–Ingelheim dihydro­pteridinone series (Lénárt *et al.*, 2007 ▸; Steegmaier *et al.*, 2007 ▸; Kothe, Kohls, Low, Coli, Rennie *et al.*, 2007 ▸; Fig. 1 ▸). For both, we determined crystal structures in parallel using both approaches. In addition, our new SER-derived crystal form of PLK1 allowed the co-crystal structure determination of the benzimidazole and clinical candidate GSK461264, which belongs to a third chemical class of PLK1 inhibitors for which no PLK1 co-crystal structure has been published to date (Fig. 1 ▸). The different binding modes of the three PLK1 lead classes will be discussed and compared.

## 2. Materials and methods

### 2.1. PLK1 SER mutant design, cloning, expression and purification

As this study was initiated when no crystal structure of the kinase domain of PLK1 was yet available, a homology model for the PLK1 kinase domain was built using the *Insight* software (Biovia) using the kinase domain of Aurora-A (PDB entry 2bmc) as the model. Predicted surface clusters of lysine and glutamate residues were identified by manual inspection, and mutations in nine separate surface clusters were designed (Table 1 ▸) employing a truncation variant of the kinase domain of human PLK1 (Gene ID 5347, transcript NM_005030.6) comprising residues Gly18–Leu340. This starting point of the current work has been described by us before (construct 5 in Fig. 1 of Bandeiras *et al.*, 2008 ▸). The mutations were introduced using the QuikChange Site-Directed Mutagenesis Kit (Agilent, Santa Clara, California, USA) and their sequences were verified. The set of PLK1 pENTRY plasmids were recombination-cloned into the previously described Gateway destination vector pD-INS1 (Bandeiras *et al.*, 2008 ▸) using Gateway LR Clonase Enzyme Mix (Invitrogen Gateway system; Thermo Fisher Scientific, Waltham, Massachusetts, USA). The resulting PLK1 SER constructs feature an N-terminal GST tag and thrombin cleavage site and were expressed in High Five insect cells using the baculovirus system as described by Bandeiras *et al.* (2008 ▸). Test expression trials identified that constructs 3, 4 and 5 yielded sufficient amounts of soluble protein. For construct 3, a large-scale expression (10 l of insect cells) was carried out and the protein was purified via glutathione Sepharose affinity chromatography (MicroSpin GST Purification Module, Amersham Biosciences catalogue No. 27-4570-03). The GST tag was cleaved overnight at 277 K on the column using human α-thrombin (catalogue No. HCT-0020-5, CellSystems). The PLK1-containing fractions were combined, concentrated and subjected to gel filtration (Superdex 75, final buffer 50 m*M* Tris–HCl pH 8.0, 300 m*M* NaCl, 10% glycerol, 5 m*M* DTT, 0.1 m*M* EDTA). The peak representing monomeric PLK1 was concentrated to 11–13 mg ml^−1^ and either used directly for crystal screening or flash-frozen in liquid nitrogen.

PLK1:DARPin co-complex preparation was carried out as described previously (Bandeiras *et al.*, 2008 ▸). In brief, a construct of PLK1 comprising residues Ala33–Lys345 (termed PLK1^WT^ hereafter) was expressed in insect cells as a GST-fusion protein, purified and cleaved via thrombin cleavage, the N-terminally His-tagged DARPin 3H10 was expressed in *Escherichia coli* and purified, and the complex of PLK1(WT) and DARPin 3H10 was formed by incubation and purified via gel filtration.

### 2.2. Small-molecule inhibitor synthesis

PLK1 inhibitor compound 1 ((2*Z*)-2-cyano-2-{3-ethyl-5-[({2-[methyl(1-methylpiperidin-4-yl)amino]pyridin-4-yl}amino)methylidene]-4-oxo-1,3-thiazolidin-2-ylidene}-*N*-(2,2,2-trifluoroethyl)acetamide) was synthesized in a seven-step synthesis route as described in the supporting information. The previously published small-molecule PLK1 inhibitors used in this study were synthesized as described in the patent applications for BI 2536 (Hoffmann *et al.*, 2004 ▸) and GSK461364 (Cheung *et al.*, 2006 ▸). BI 2536 and GSK461364 are now also available from commercial vendors.

### 2.3. PLK1 activity assay

The inhibitory activities of the compounds against PLK1 were measured in a TR-FRET-based kinase-activity inhibition assay using the biotinylated peptide biotin-Ahx-KKLNRTLSFAEPG (C-terminus in amide form, Biosyntan) as a substrate. The recombinant kinase-domain construct PLK1^WT^ (residues 33–345, expressed and purified as described in Bandeiras *et al.*, 2008 ▸) was used as the enzyme. In brief, a recombinant fusion protein comprising an N-terminal GST tag, a thrombin cleavage site (AAAPFTLVPRGS) and the PLK1^WT^ kinase domain was expressed in baculovirus-infected insect cells (High Five) and bound to glutathione Sepharose. After a washing step, the PLK1 kinase domain was released from the glutathione Sepharose by incubation with thrombin and purified by size-exclusion chromatography.

For the assay, 50 nl of a 100-fold concentrated solution of the test compound in DMSO was pipetted into a black low-volume 384-well microtitre plate (Greiner Bio-One), 2 µl of a solution of PLK1 (final concentration 0.015 ng µl^−1^) in aqueous assay buffer [50 m*M* HEPES pH 7.0, 25 m*M* MgCl_2_, 1.0 m*M* dithiothreitol (DTT), 0.05%(*w*/*v*) bovine serum albumin (BSA), 0.001%(*v*/*v*) Nonidet-P40 (Sigma), 1× Complete EDTA-free protease-inhibitor mixture (Roche)] was added and the mixture was incubated for 15 min at 22°C to allow pre-binding of the test compounds to the enzyme before the start of the kinase reaction. The kinase reaction was started by the addition of 3 µl of a solution of ATP (final concentration 10 m*M*) and substrate (final concentration 0.84 µ*M*) in assay buffer and the resulting mixture was incubated for a reaction time of 30 min at 22°C. The reaction was stopped by the addition of 5 µl of a solution of TR-FRET detection reagents [0.4 µ*M* streptavidin XL665 (Cisbio Bioassays) and 1 n*M* anti-phosphoserine antibody (Merck Millipore, catalogue No. 35-002) and 1.5 n*M* LANCE Eu-W1024 labelled anti-mouse IgG antibody (Perkin-Elmer, product No. AD0077)] in an aqueous EDTA solution [100 m*M* EDTA, 0.12%(*w*/*v*) bovine serum albumin in 100 m*M* HEPES pH 7.5].

The resulting mixture was incubated for 1 h at 22°C to allow the formation of a complex between the phosphorylated biotinylated peptide and the detection reagents. Subsequently, the amount of phosphorylated substrate was evaluated by measurement of the resonance energy transfer from the europium chelate to the streptavidin XL665. The fluorescence emission at 620 and 665 nm after excitation at 337 nm was measured in a Pherastar FS (BMG Labtechnologies). The ratio of the emission at 665 and 620 nm was taken as a measure of the amount of phosphorylated substrate. The compounds were tested on the same microtitre plate at 11 different concentrations in the range 20 µ*M* to 0.1 n*M* in duplicate for each concentration and IC_50_ values were calculated by a four-parameter fit.

### 2.4. Crystallization, data collection and structure determination

Crystals of the PLK1^WT^:DARPin complex were obtained as described in Bandeiras *et al.* (2008 ▸). For inhibitor co-complex formation, crystals were soaked for 24 h with 2 m*M* inhibitor, then cryoprotected using a stepwise approach in a mother-liquor solution containing increasing glycerol concentrations of 5, 15 and 25%(*v*/*v*), each for 5 min, followed by flash-cooling in liquid nitrogen. Diffraction data were collected on the ESRF beamlines ID14-1 and ID29 in Grenoble and integrated with *XDS* (Kabsch, 2010 ▸). Structures were solved using *Phaser* (McCoy *et al.*, 2007 ▸) with an inhibitor-free PLK1^WT^:DARPin co-crystal structure (PDB entry 2v5q) as a search model, refined using *REFMAC*5 (Kovalevskiy *et al.*, 2018 ▸) and rebuilt using *Coot* (Emsley *et al.*, 2010 ▸). The data-collection statistics and refinement statistics are shown in Tables 2 ▸ and 3 ▸.

Initial crystallization conditions for the kinase domain of PLK1 without a DARPin (residues 18–340 with mutations K225D and K226A, termed PLK1^K225D/K226A^ hereafter) were identified in sparse-matrix screens using the hanging-drop method. Crystals were obtained with a reservoir consisting of 1.7 *M* ammonium sulfate, 0.1 *M* MES pH 6.5, 5% ethanol. Optimized crystals were obtained in drops made from 1 µl reservoir solution (0.1 *M* sodium acetate, 1.68–2.10 *M* ammonium sulfate, 0.1 *M* MES pH 6.5) and 1 µl protein solution (11–13.5 mg ml^−1^ in gel-filtration buffer). For ligand co-crystallization, PLK1 was incubated for 1–2 h with 2 m*M* ligand (diluted from 100 m*M* stock solution in DMSO) at 277 K. Crystals were grown at 293 K. They were cryoprotected by a brief exposure to cryo-buffer [2 *M* ammonium sulfate, 0.1 *M* MES pH 6.5, 0.1 *M* sodium acetate, 20%(*v*/*v*) glycerol, 1 m*M* ligand, 1% DMSO]. Diffraction data were collected on beamline 14.2 at the BESSY II electron-storage ring operated by the Helmholtz-Zentrum Berlin (Mueller *et al.*, 2015 ▸) and processed using *HKL*-2000 (Otwinowski & Minor, 1997 ▸). The structure with compound 1 was solved by molecular replacement using *MOLREP* (Vagin & Teplyakov, 2010 ▸) from the *CCP*4 program suite (Agirre *et al.*, 2023 ▸) with PLK1 from the DARPin co-crystal structure (PDB entry 2v5q) as a search model. Subsequent co-complex structures were solved using rigid-body refinement in *REFMAC*5 (Kovalevskiy *et al.*, 2018 ▸). Ligand parameters were calculated using *PRODRG* (Schüttelkopf & van Aalten, 2004 ▸). The structures were refined and rebuilt using *REFMAC*5 and *Coot* (Emsley *et al.*, 2010 ▸). Data-collection and refinement statistics are shown in Tables 2 ▸ and 3 ▸.

## 3. Results

### 3.1. SER mutant design, structure determination and effect of the SER mutation

The SER approach postulates that when residues with many rotatable bonds, such as lysine, arginine, glutamic acid and glutamine, are located on the surface of a protein they can adopt many different alternative rotamer conformations and thus possess high surface entropy. If forced to contribute to a crystal contact, hydrogen bonds and salt bridges formed by these residues within the crystal contact will contribute favourably to the enthalpy term of the free enthalpy of crystal growth. However, this is counterbalanced by the loss of surface entropy when these residues are frozen into one conformation in the new contact. If several such residues with highly entropic side chains form one continuous surface epitope, either by direct proximity in the primary sequence or by proximity caused by the protein fold, these patches of high surface entropy may prevent crystal contact formation altogether. The art of SER mutant design is to (i) identify such surface clusters (either in a structure if a structure is already available, or in a homology model or *AlphaFold* model if no structure has yet been solved) and (ii) to choose suitable amino acids with less surface entropy that can be accommodated in the structure and do not negatively impact the folding of the protein. As no PLK1 structure had yet been published when this work was initiated and *AlphaFold* was not yet available, surface clusters of lysine and glutamate residues were identified in a homology model. The surface of the homology model was manually inspected, and surface clusters were identified where several lysine and/or glutamate residues formed one continuous surface epitope, and their side chains were clearly directed into the solvent and were not contributing to the overall kinase fold or to the ATP-binding site. The nine clusters of SER mutations selected by this procedure are listed in Table 1 ▸. In this project, we decided to focus only on glutamate and lysine residues and to mutate them not exclusively to alanine but mainly to aspartate in order to conserve their polar nature and, in the case of glutamate, their negative charge (Table 1 ▸).

The nine SER mutants were introduced into a PLK1 construct comprising residues 18–340. This PLK1 truncation variant had been identified previously by limited proteolysis (Bandeiras *et al.*, 2008 ▸) and, like the wild-type construct, expressed well in High Five insect cells and was well folded as judged from ITC curves for the interaction with staurosporine (data not shown), but did not crystallize. Initial expression tests indicated that three of the nine SER mutants, constructs 3, 4 and 5 (Table 1 ▸), could be expressed as soluble proteins using High Five insect-cell expression.

**Figure 2:**
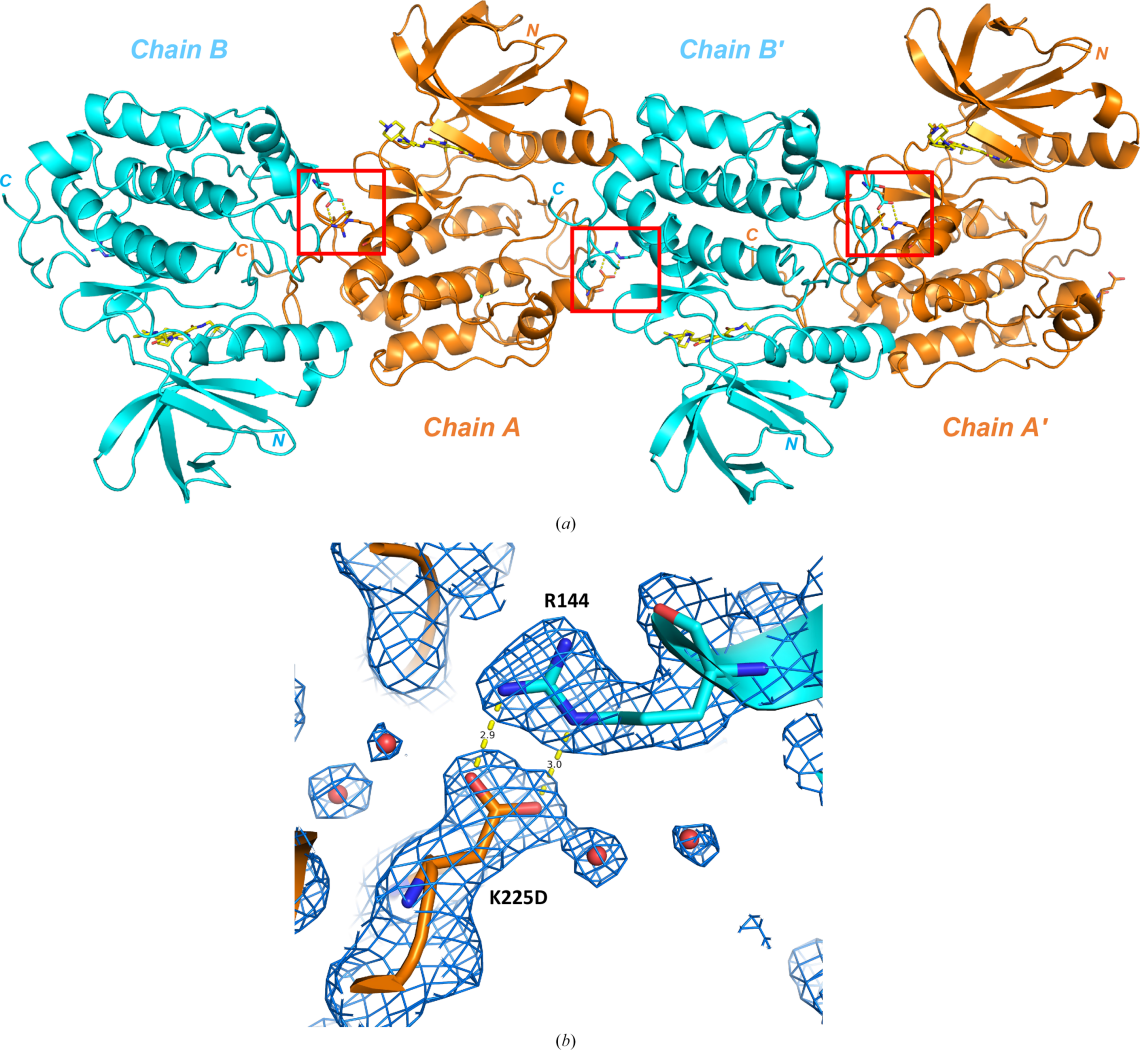
SER mutation K225D enables a crystal-packing contact. (*a*) The noncrystallographic PLK1^K225D/K226A^ dimer in the asymmetric unit (chains *A* and *B*) and a crystal neighbour (chains *A*′ and *B*′) are shown in cartoon representation. Residue Arg144 and the SER mutation K225D are shown in stick representation. The crystal contacts formed by a salt bridge between Arg144 and the SER mutation K225D are highlighted by red boxes. (*b*) shows a zoom into this double salt bridge (2*F*_o_ − *F*_c_ density map at 2.2 Å resolution, contoured at 1.3σ; hydrogen bonds shown as yellow dotted lines).

Of these three soluble SER variants, the double mutant of construct 3, PLK1^K225D/K226A^, was expressed and purified on a larger scale and subjected to crystallization screening. PLK1^K225D/K226A^ crystallized readily under several conditions in the initial screen. Optimized conditions resulted in a well reproducible crystallization system, yielding crystals that typically diffracted to between 2.2 and 2.8 Å resolution, which were subsequently used for co-crystallization studies. The asymmetric unit contains two PLK1 molecules, chain *A* and chain *B*, as illustrated by the co-crystal structure with compound 1 (Fig. 2 ▸*a*). In both chains, the electron-density map showed clear density for the side chains of the mutated surface residues Asp225 and Ala226 (Asp225 is shown in Fig. 2 ▸*b*). Crystal-packing analysis revealed that Asp225 from the newly introduced K225D mutation enabled the formation of a crystal contact. The side chain of Asp225 in chain *B* forms a salt bridge to the side chain of Arg144 from chain *A*, the second PLK1 monomer in the asymmetric unit. This inter­action pattern is repeated by the side chain of Asp225 of chain *A*, which forms the same salt bridge with Arg144 from a crystallographically related chain *B*′ (Fig. 2 ▸*a*).

In this structure and in all further inhibitor co-complex structures of PLK1^K225D/K226A^ presented here, the PLK1 kinase domain crystallized in the active conformation as judged from the DFG-in conformation and the presence of a salt bridge between Lys82 in the ATP-binding site and Glu101 from helix *C*. Also, in this and all further inhibitor co-complex crystal structures of PLK1^K225D/K226A^ reported here, a sulfate ion from the crystallization buffer is bound adjacent to Thr210 in the activation loop and a disulfide bridge is observed between Cys255 in the *A* chain and the same Cys255 in a crystallographically related chain *A*′ of a crystal neighbour. The sulfate ion mimics the phosphoryl moiety of PLK1 in the active state (phosphorylated at Thr210), as also observed in the crystal structure of fully activated zebrafish PLK1 reported by Elling *et al.* (2008 ▸) (PDB entries 3d5w and 3d5x). The disulfide bond is most likely a crystallization artefact triggered by the proximity of the two residues in the crystal lattice.

### 3.2. Binding mode of thiazolidinone compound 1

To reveal the PLK1 binding pocket and to understand the molecular interactions that small-molecule inhibitors can form within this pocket, we determined co-crystal structures with representatives of three different inhibitor classes. The chemical structures are shown in Fig. 1 ▸. We determined IC_50_ values using an HTRF-based kinase-activity assay. In the cell, an ATP-site kinase inhibitor must compete against the high endogenous concentration of ATP, which is in the range 1–5 m*M* (Greiner & Glonek, 2021 ▸). To mimic this situation and to resolve the potencies of very potent project compounds and tool compounds, the ATP background concentration in the kinase assay is often increased to 1 m*M* [see, for example, the inhibitor-optimization campaigns for the kinases TBK1 (Lefranc *et al.*, 2020 ▸) and IRAK4 (Bothe *et al.*, 2024 ▸)] or to 2 m*M* (see kinase MPS1; Schulze *et al.*, 2020 ▸). For PLK1 we increased the ATP concentration to 10 m*M*. Table 4 ▸ shows the IC_50_ values determined in this study for the published thiazolidinone inhibitor TAL (Santamaria *et al.*, 2007 ▸) and for the three inhibitors for which we determined co-crystal structures with PLK1. While TAL shows an IC_50_ value of only 184 n*M* under these high-ATP assay conditions (compared with 19 ± 12 n*M* at 0.5 µ*M* ATP; Santamaria *et al.*, 2007 ▸), the further optimized thiazolidinone compound 1 shows an IC_50_ value of 4.3 n*M* and the other two optimized PLK1 inhibitors, BI 2536 and GSK461364, also feature IC_50_ values in the single-digit nanomolar range. The IC_50_ value determined for GSK461364 is in line with published data (apparent dissociation constant 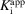 < 0.5 n*M*; Gilmartin *et al.*, 2009 ▸) when taking into account the higher ATP concentration in our assay, and confirmed its high potency.

#### 3.2.1. Co-crystal structures of thiazolidinone compound 1 via the surface-entropy reduction and DARPin approaches

**Figure 3:**
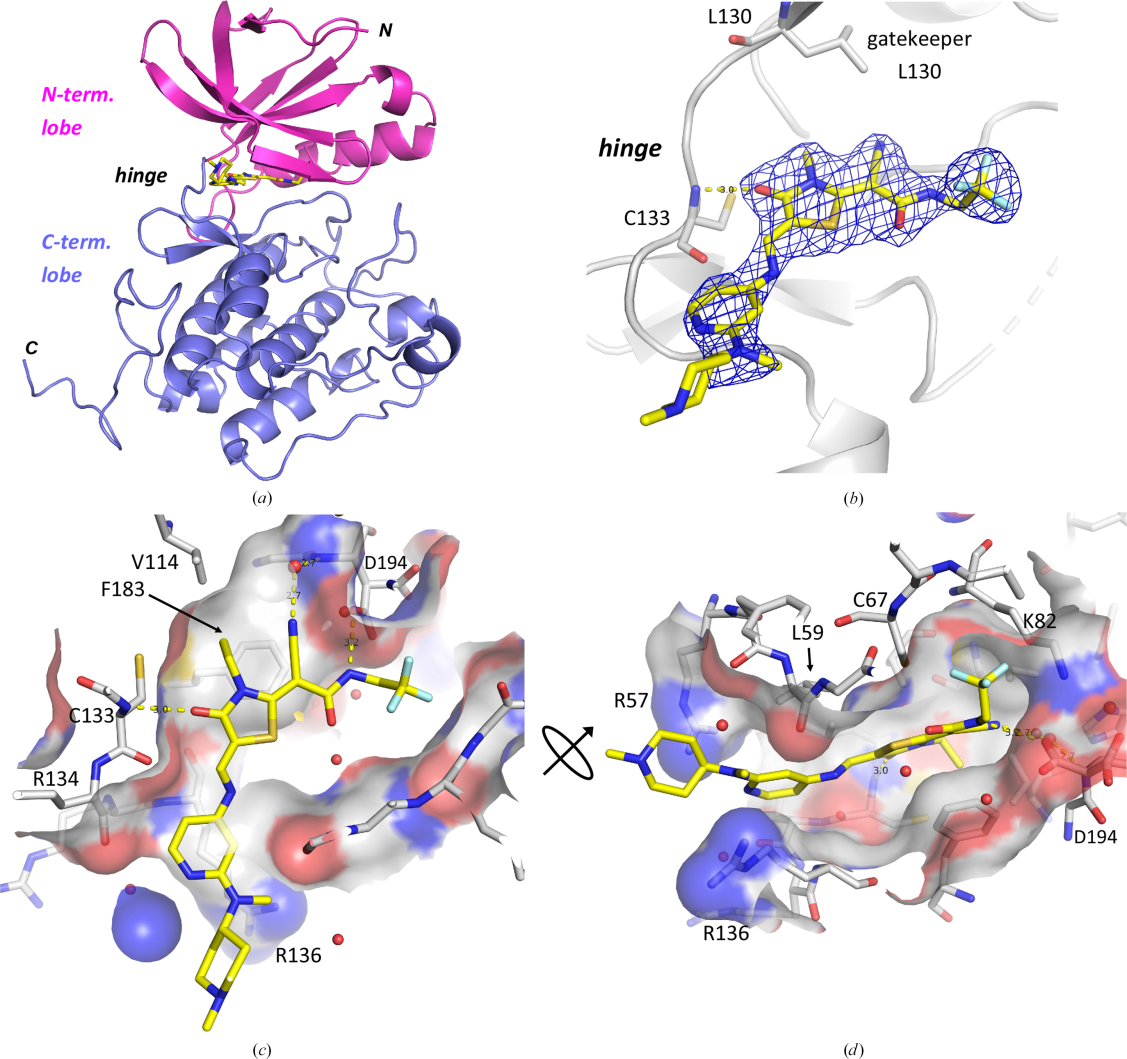
Compound 1 co-crystallized with the SER mutant PLK1^K225D/K226A^. (*a*) shows an overall view of the kinase domain of PLK1^K225D/K226A^ (compound 1 in stick representation, C atoms in yellow). Chain *A* of the two PLK1 chains in the asymmetric unit is shown (PDB entry 9r1w). (*b*) 2*F*_o_ − *F*_c_ density map contoured at 1.3σ, with Cys133 at the hinge of PLK1 and the gatekeeper residue Leu130 shown in stick representation for orientation. (*c*) and (*d*) show a view into the ATP-binding pocket of PLK1, with PLK1 in surface representation. PLK1 residues lining the inhibitor-binding site are shown with C atoms in white and in stick representation. PLK1 is depicted with a semi-transparent surface.

Compound 1 of the thiazolidinone inhibitor class was co-crystallized with the SER mutant PLK1^K225D/K226A^. Fig. 3 ▸(*a*) shows the overall structure of PLK1 chain *A*. (The ligand-binding mode in chains *A* and *B* is the same. As the ligand in chain *A* features lower *B* factors and is better defined in the electron density, the following analysis will focus on the binding event in chain *A*, unless otherwise stated.) PLK1 features the typical bilobal overall fold of a protein kinase domain, with the ATP-binding pocket located between these two lobes in the hinge region (Fig. 3 ▸*a*). Figs. 3 ▸(*a*) and 3 ▸(*b*) show that compound 1 binds within this ATP-binding site at the hinge region (type I kinase inhibitor). The hinge region in PLK1, with the central residue Cys133, offers three polar interaction points for ATP-site kinase inhibitors: the backbone nitrogen and carbonyl group of Cys133, of which compound 1 targets the backbone nitrogen (Fig. 3 ▸*b*), and the backbone carbonyl group of Glu131 (which is not addressed by a classical hydrogen bond to compound 1; instead see Section 4.4, Fig. 8). The residue immediately preceding the hinge region (here Leu130; Fig. 3 ▸*b*) is termed the gatekeeper residue. If a protein kinase features a residue with a small side chain (typically threonine or valine), it allows ATP-site binders to insert deeper into this section of the ATP site. If this residue is larger (for example the leucine residue in PLK1), it closes the gate to the back pocket.

On the ligand side, the thiazolidinone ring acts as the hinge-binding motif. It interacts via its carbonyl oxygen with the backbone nitrogen of Cys133 of the hinge region. The ethyl moiety of this ring fills a small hydrophobic pocket under the gatekeeper Leu130 and adjacent to the residues Val114 and Phe183 and donates a nonclassical or weak C—H⋯O hydrogen bond to Glu131 in the hinge region. The nitrile group accepts a water-bridged hydrogen bond from the backbone amide nitrogen of Asp194 of the DFG motif. The side chain of this aspartate accepts another hydrogen bond, from the amide nitrogen of compound 1. The S atom of the thiazolidinone ring is in direct van der Waals contact with the S atom of Cys67 in the roof of the ATP site (S–S distance 4.0 Å). The other end of the elongated ligand points towards the solvent. Here, the outer pyridine ring donates two further nonclassical C—H⋯O=C hydrogen bonds to the backbone carbonyl atoms of Cys133 (3.3 Å) and Arg134 (3.4 Å). The structure reveals that the C=C double bond between the thiazolidinone ring and the outer pyridino-aniline nitrogen is in the *Z* conformation when compound 1 is bound to PLK1. In solution, this double bond is assumed to exchange between the *E* and *Z* conformation. The pyridine ring and the outer methylamino group pack between the side chains of Arg136 on the floor and Leu59 in the roof of the ATP pocket (Figs. 3 ▸*c* and 3 ▸*d*). The pyridine nitrogen and the outer piperidine ring stack against the guanidinium head group of Arg57.

**Figure 4:**
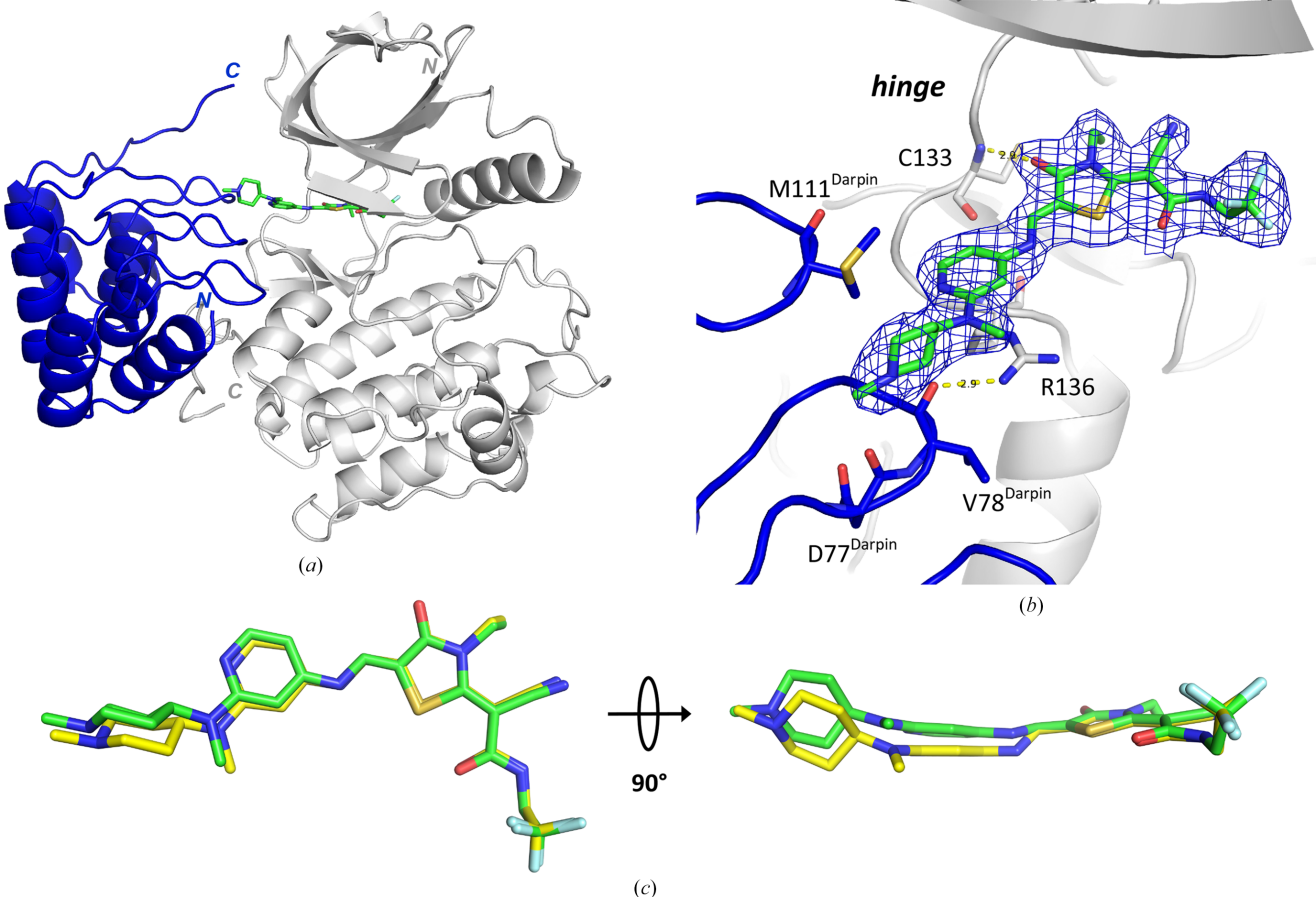
Binding mode of thiazolidinone compound 1 soaked into a crystal of PLK1^WT^:DARPin. (*a*) Overall view of the complex of the kinase domain of PLK1^WT^ (grey ribbon representation), the DARPin molecule (blue ribbon representation) and compound 1 (stick representation, C atoms in green). Hetero­dimer 1 (chains *A* and *C*) of the two dimers in the asymmetric unit is shown (PDB entry 9r8b). (*b*) 2*F*_o_ − *F*_c_ density map contoured at 1.3σ, with Cys133 in the hinge of PLK1 and the DARPin residues Asp77, Val78 and Met111 (in contact with the ligand) shown in stick representation. Hydrogen bonds are shown as dotted yellow lines. (*c*) Overlay of the binding modes of compound 1 from the SER approach (chain *A*, yellow C atoms) and from the DARPin approach (chain *A*, green C atoms), obtained by superimposition of the respective protein chains.

In parallel to our SER campaign, we also soaked compound 1 into crystals of the PLK1^WT^:DARPin complex and were able to determine the co-crystal structure with compound 1 at 2.2 Å resolution (Figs. 4 ▸*a* and 4 ▸*b*). This outcome confirmed that the ATP-binding site was still accessible for ATP-site inhibitors within the PLK1:DARPin crystal lattice. The binding mode observed here is in principle identical to that observed upon co-crystallization with PLK1^K225D/K226A^. A superimposition of both structures (Fig. 4 ▸*c*) shows that the binding poses within the ATP-binding pocket are the same for the DARPin approach and for the SER approach, and the inhibitor double bond in the DARPin-derived structure is again found to be in the *Z* configuration. There are small differences in the outer part of the ligand, which protrudes from the ATP site towards the solvent. Here, the outer pyridine and aminopiperidine moieties stretch out and stack against the side chain of Arg136 at the lower entrance to the ATP-binding site (Figs. 3 ▸*c* and 4 ▸*b*). In the DARPin structure, Arg136 engages in a hydrogen bond to the backbone carbonyl O atom of Val78^DARPin^, and the residues Asp77^DARPin^, Val78^DARPin^ and Met111^DARPin^ are in contact with the pyridine ring and the outer aminopiperidine moiety of the ligand. Overall, these interactions of the DARPin with the outer part of the elongated ligand result in a slight tilt of the outer pyrimidine and piperidine units in the DARPin complex when it is compared with the PLK1^K225D/K226A^ structure (Fig. 4 ▸*c*).

#### 3.2.2. Probing the two crystallization approaches with the tool compound BI 2536

**Figure 5:**
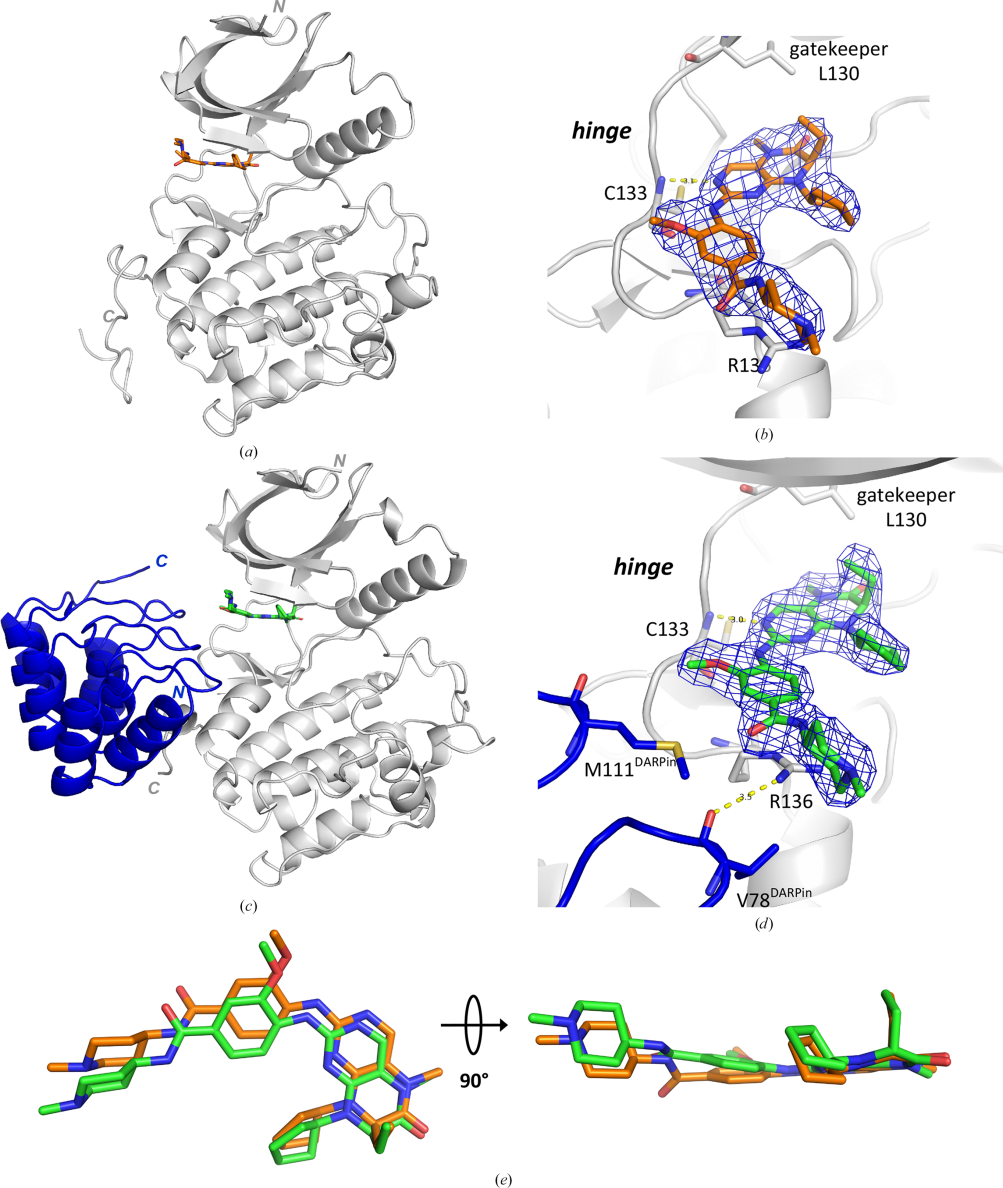
Binding mode of BI 2536 in the ATP-binding site of PLK1^K225D/K226A^ (SER approach) and in PLK1^WT^ in complex with the DARPin. (*a*) Overall view of the complex of PLK1^K225D/K226A^ (grey ribbon) and BI 2536 (in stick representation with C atoms in orange; PDB entry 9r1x). (*b*) 2*F*_o_ − *F*_c_ density map contoured at 1.3σ, with Cys133 in the hinge region and the gatekeeper residue Leu130 shown in stick representation. Hydrogen bonds are shown as dotted yellow lines. (*c*) Overall view of PLK1^WT^ (grey ribbon representation), the DARPin (blue ribbon representation) and BI 2536 (stick representation, C atoms in green; PDB entry 9r8c). Heterodimer 2 (chains *B* and *C*) is shown of the two dimers in the asymmetric unit. (*d*) 2*F*_o_ − *F*_c_ density map contoured at 1.3σ, with PLK1 residues Cys133 (hinge) and Leu130 (gatekeeper) and DARPin residues Val78 and Met111 (in contact with the ligand) shown in stick representation. (*e*) Overlay of BI 2536 from both approaches, obtained by superimposition of the protein chains (chain *A* of the PLK1^K225D/K226A^ structure and chain *B* of the DARPin structure).

The dihydropteridinone compound BI 2536 (Fig. 1 ▸*c*) has been tested in clinical trials (Frost *et al.*, 2012 ▸; Mross *et al.*, 2012 ▸) and is a precursor of the clinical candidate Volasertib (BI-6727; Rudolph *et al.*, 2009 ▸). We used it here as a tool compound to further compare the two different PLK1 crystallization approaches. Figs. 5 ▸(*a*) and 5 ▸(*b*) show this compound co-crystallized with PLK1^K225D/K226A^, solved at 2.8 Å resolution. Figs. 5 ▸(*c*) and 5 ▸(*d*) show the same inhibitor bound to PLK1^WT^ after soaking into PLK1^WT^:DARPin crystals, solved at 2.5 Å resolution. In addition, this inhibitor has also been co-crystallized in complex with PLK1^T210V^ (PDB entry 2rku; Kothe, Kohls, Low, Coli, Rennie *et al.*, 2007 ▸). Both of the two PLK1 chains of the PLK1^K225D/K226A^:BI 2536 structure and of the PLK1^WT^:DARPin:BI 2536 structure superimpose very well with the PLK1^T210V^:BI 2536 structure, with r.m.s.d. values over all C^α^ atoms of between 0.60 and 0.71 Å. In all structures, the inhibitor is bound in the same binding mode (shown for the SER variant and the DARPin complex in Fig. 5 ▸*e*). However, while the section of the inhibitor bound deeply in the ATP pocket always shows the same binding pose, the outer, more solvent-exposed piperidine moiety in the DARPin co-complex deviates slightly in its position. The plane of the inhibitor, comprising of the coplanar dihydropteridinone and aminophenyl ring systems and the outer amide group, is tilted by about 5° within the ATP-binding pocket when comparing the DARPin structure with the SER approach (Fig. 5 ▸*e*) and with the T210V approach (not shown in Fig. 5 ▸*e*). This tilt of the inhibitor within the binding site is most likely caused by the presence of the DARPin, which contacts PLK1 immediately in front of the ATP site. As observed before with compound 1 (Fig. 4 ▸*b*), the backbone carbonyl O atom of Val78^DARPin^ accepts a hydrogen bond from Arg136 at the lower entrance of the ATP-binding site and thereby affects its rotamer conformation when compared with the DARPin-free structures, and the side chain of Met111^DARPin^ stacks against the outer part of the ligand (Fig. 5 ▸*d*).

#### 3.2.3. Binding mode of the PLK1 inhibitor and clinical candidate GSK461364

**Figure 6:**
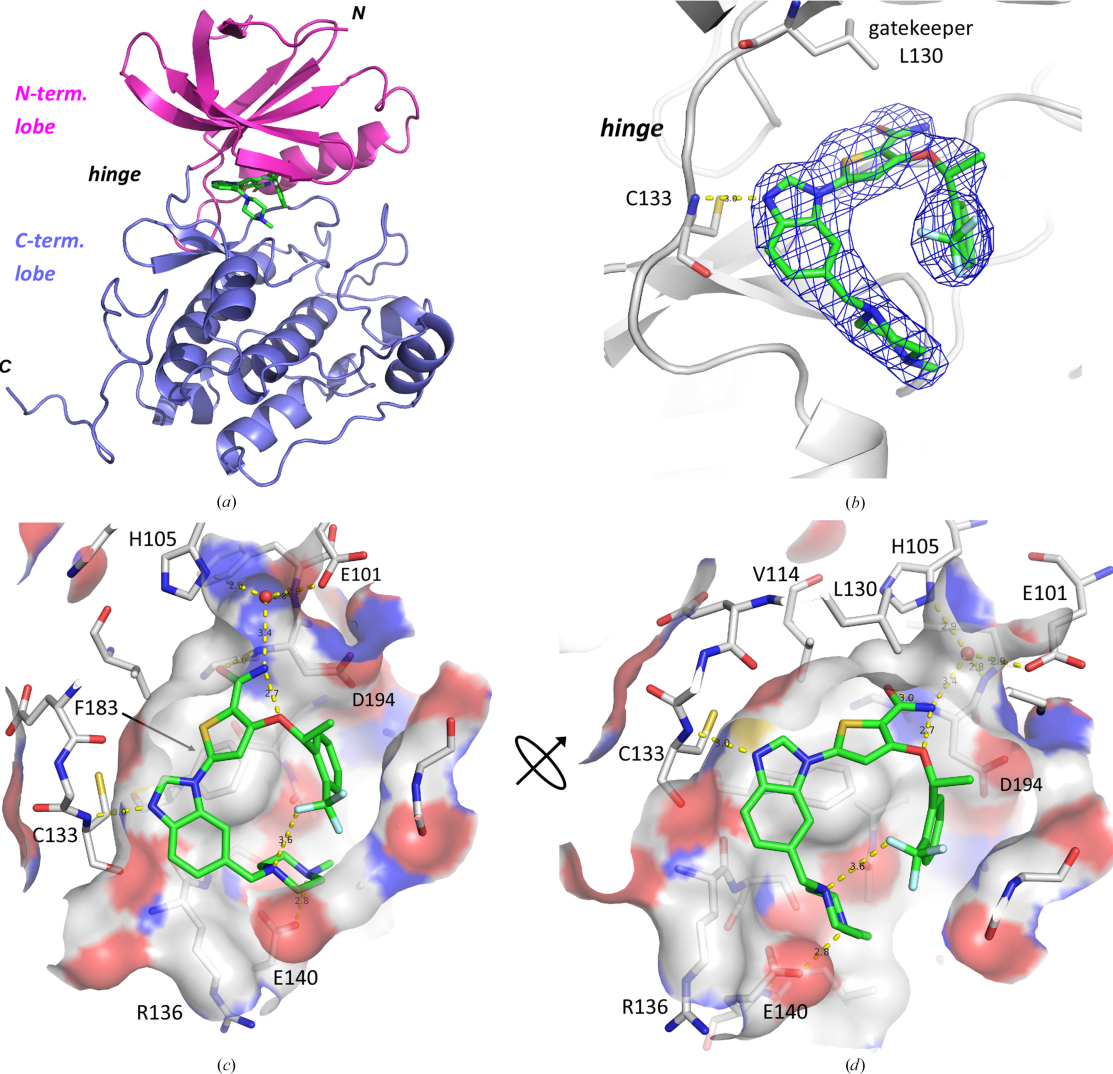
Binding mode of benzimidazole GSK461364 in the ATP-binding site of PLK1^K225D/K226A^. (*a*) Overall view, with PLK1^K225D/K226A^ as a ribbon model and GSK461364 in stick representation (C atoms in green; PDB entry 9r1y). (*b*) 2*F*_o_ − *F*_c_ density map contoured at 1.3σ, with Cys133 at the hinge of PLK1 and the gatekeeper residue Leu130 shown in stick representation. Hydrogen bonds are shown as dotted yellow lines. (*c*, *d*) View into the ATP-binding site, hinge region on the left; (*d*) shows a rotated view. PLK1 residues are shown as grey C atoms with a semi-transparent molecular surface.

The benzimidazole compound GSK461364 is a potent inhibitor of PLK1 and has been tested in clinical phase I trials in patients with advanced solid malignancies (Olmos *et al.*, 2011 ▸). The co-complex structure with PLK1^K225D/K226A^ (Fig. 6 ▸) confirms that it binds to the ATP-binding site. It interacts with the hinge region via one hydrogen bond formed between the free imidazole nitrogen and the backbone amide of Cys133. The thiophene ring inserts under the gatekeeper residue Leu130 and forms a π–π stacking interaction with Phe183. The amide group of the inhibitor is engaged in a network of hydrogen bonds in the depths of the ATP-binding site. The amide O atom accepts a direct hydrogen bond from the backbone amide N atom of Asp194 of the DFG motif. The amide nitrogen of GSK461364 donates a hydrogen bond to a water molecule, which in turn is coordinated by the side chains of His105 and of the conserved Glu101 of helix C, as well as by the backbone nitrogen of Phe195 of the DFG motif. The outer piperazine ring donates a hydrogen bond to the side chain of Glu140 in front of the ATP-binding pocket. The trifluoro­methyl group points upwards towards the P loop and fills a subpocket in the roof of the ATP-binding site. An F atom of the trifluoromethyl group accepts an intramolecular N—H⋯F hydrogen bond (3.6 Å) from an N atom of the piperazine ring of the inhibitor (Figs. 6 ▸*c* and 6 ▸*d*). Similarly, the amide nitrogen of the inhibitor is engaged in another intramolecular hydrogen bond to the adjacent ether oxygen. These intramolecular hydrogen bonds may already favourably stabilize the inhibitor in solution in the horseshoe-shaped overall conformation that is observed here upon binding into the ATP-binding pocket of PLK1.

## 4. Discussion

### 4.1. Lessons on SER mutant design

Despite intensive crystal-screening efforts, neither the wild-type version of the PLK1 truncation variant used as the basis for our SER mutation PLK1^K225D/K226A^ nor the wild-type version of the slightly shorter PLK1 truncation variant used for crystallization with the DARPin produced any crystals. In contrast, but in line with the concept of the surface-entropy reduction (SER) approach, the SER mutant PLK1^K225D/K226A^ crystallized readily under several conditions in the first sparse-matrix screen. The SER mutations introduced in this study had been chosen to replace predicted surface lysine and glutamate residues with residues with shorter and more rigid side chains (Table 1 ▸). In the early days of the development of the SER approach, predicted lysine and glutamate surface residues were mutated exclusively to alanine (reviewed in Derewenda, 2004 ▸). For PLK1 we explicitly did not mutate all lysine and glutamate residues in the predicted surface clusters to alanine, but instead relied heavily on mutations to aspartate (Table 1 ▸). This was done to reduce the surface entropy while retaining the polar and charged character, as we expected that additional alanine surface residues would increase the hydrophobicity and reduce protein solubility. Still, only three of the nine designed SER mutations resulted in soluble protein variants (Table 1 ▸), and these have in common that they feature a maximum of two or three point mutations. The SER mutants which consisted of four and five point mutations all turned out to be insoluble. In hindsight, introducing so many aspartate residues and thus negative charges in places which before had predominantly featured positively charged lysine residues may have been too drastic a change. Also, mutating more than three neighbouring PLK1 residues simultaneously may have had an unfavourable effect on proper folding. In line with this, Cooper and coworkers also suggested mutating predicted high-entropy surface resides not only to alanine but to other rigid polar residues such as threonine (Cooper *et al.*, 2007 ▸), and we have recently reported successful SER approaches for the RAS exchange factor SOS2 (Hillig *et al.*, 2019 ▸) and the protein kinase BUB1 (Schaefer *et al.*, 2024 ▸), where mutations to tyrosine residues enabled crystallization. In all cases, it was important to test a larger set of SER mutants to increase the likelihood of finding mutants that are still expressed as soluble protein and can be tested in crystallization screens.

### 4.2. Comparison of the DARPin- and SER-enabled crystal structures of PLK1

**Figure 7:**
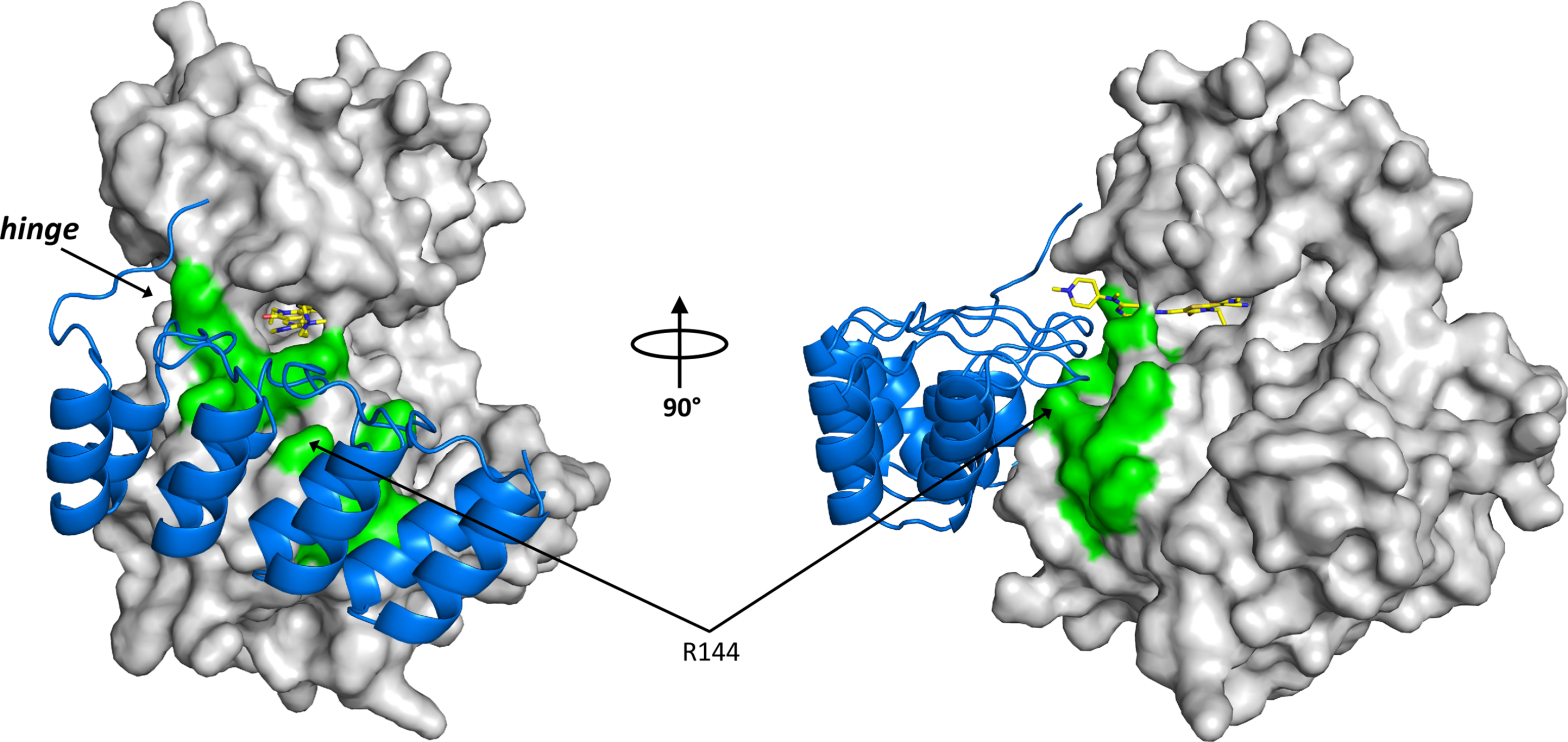
The DARPin binding epitope covers the nuclear localization signal (NLS) of PLK1. The DARPin complex of PLK1^WT^ in complex with compound 1 is shown (PDB entry 9r8b; chains *A* and *C*, with PLK1 as a grey surface, the DARPin in blue ribbon representation and compound 1 in stick representation with yellow C atoms). The residues ^134^RRR^136^ and ^143^KRRK^146^ of the NLS sequence are highlighted as green surface patches. This NLS epitope also comprises residue Arg144 which, in the SER approach, is engaged in the crystal contact formed with the SER point mutation K225D.

The choice of introducing one alanine and one aspartate in our successful K225D/K226A double mutant did not only produce soluble protein, but serendipitously also enabled the formation of a crystal contact. The side chain of Asp225 from the SER mutation approach forms a salt bridge and accepts a double hydrogen bond from Arg144 in chain *B* from the second PLK1 monomer in the asymmetric unit and in chain *A* from the next crystallographic neighbour (Fig. 2 ▸). Further analysis of this contact revealed that the counterpart of Asp225, residue Arg144 from the crystal neighbour, belongs to a larger, highly entropic surface cluster (^134^RRR^136^ and ^143^KRRK^146^) with a total of five arginine and two lysine residues (Fig. 7 ▸). Indeed, we had predicted this group of basic residues to form a high-entropy surface cluster in our PLK1 homology model and had tried to target it with SER construct 6 (K143D/K146D), but this construct did not produce sufficient soluble protein in expression trials. This Arg- and Lys-rich surface area had earlier been identified as the nuclear localization signal (NLS) that targets PLK1 to the nucleus (Taniguchi *et al.*, 2002 ▸). Comparison with the DARPin co-crystal structures reported here revealed that this same surface cluster targeted by Asp225 of the SER mutant is also targeted by the DARPin, which shields and masks this highly entropic surface (Fig. 7 ▸). The DARPin forms salt bridges to four of the six arginine residues and to one of the two lysine residues of the NLS sequence and, in addition, also interacts with two further adjacent lysine residues and two glutamate residues (see Supplementary Table S1). The DARPin approach and the SER approach thus probably facilitated PLK1 crystallization via the same principle: by finding a way to engage an Arg- and Lys-rich surface patch in an interaction, which reduced or masked its detrimental effect on crystal-contact formation.

A comparison of the corresponding pairs of crystal structures of identical ligands, crystallized via the SER approach and via the DARPin approach, revealed that the binding modes within the ATP pocket and the crucial interactions with the hinge region were fully consistent with each other. Small deviations were observed in the outer, more solvent-exposed parts of the ligands, where the immediately adjacent DARPin appears to have slightly modified the binding conformation of the ligand. In summary, we evaluate both approaches as valid approaches to determine the binding modes of new chemical classes of PLK1 small-molecule inhibitors. Considering the reduced amount of work to produce just one protein (in the case of the PLK1 SER variant) versus a complex of two proteins (in the case of the DARPin complex) and taking into consideration the small artificial effects of the DARPin on the solvent-exposed ligand regions, the PLK1 SER variant identified in this study represents an excellent new crystallization platform for PLK1–inhibitor co-crystal structures.

### 4.3. All PLK1 crystallization approaches required surface modifications

Remarkably, all reported crystallization strategies for PLK1 required modification of the protein surface. The first structure of PLK1, published by Kothe and coworkers from Pfizer, used the T210V mutation; Thr210 is the position within the activation segment of PLK1 where it is phosphorylated by an upstream kinase (Kothe, Kohls, Low, Coli, Cheng *et al.*, 2007 ▸). Mutating this residue to valine ensured homogenously non­phosphorylated PLK1 from insect-cell expression. However, even with this surface mutation in place, optimization of the corresponding crystal form required a very lengthy optimization process and an unusually low concentration of zinc ions in the reservoir buffer. Elling and coworkers at Sunisis reported that human wild-type PLK1 did not crystallize, and they had to switch to zebrafish PLK1, making use of the surface modification introduced during the divergent evolution between *Homo sapiens* and this fish ortholog (Elling *et al.*, 2008 ▸), while validating this ortholog approach by testing a large number of inhibitors of human PLK1 for activity against zebrafish PLK1. Finally, Beria and coworkers at Nerviano obtained crystals of human PLK1 only after post-translational lysine methylation (Beria *et al.*, 2010 ▸), *i.e.* employing an alternative route to our SER mutation approach to modify surface lysine residues.

### 4.4. Binding modes of the different PLK1 inhibitor classes

**Figure 8:**
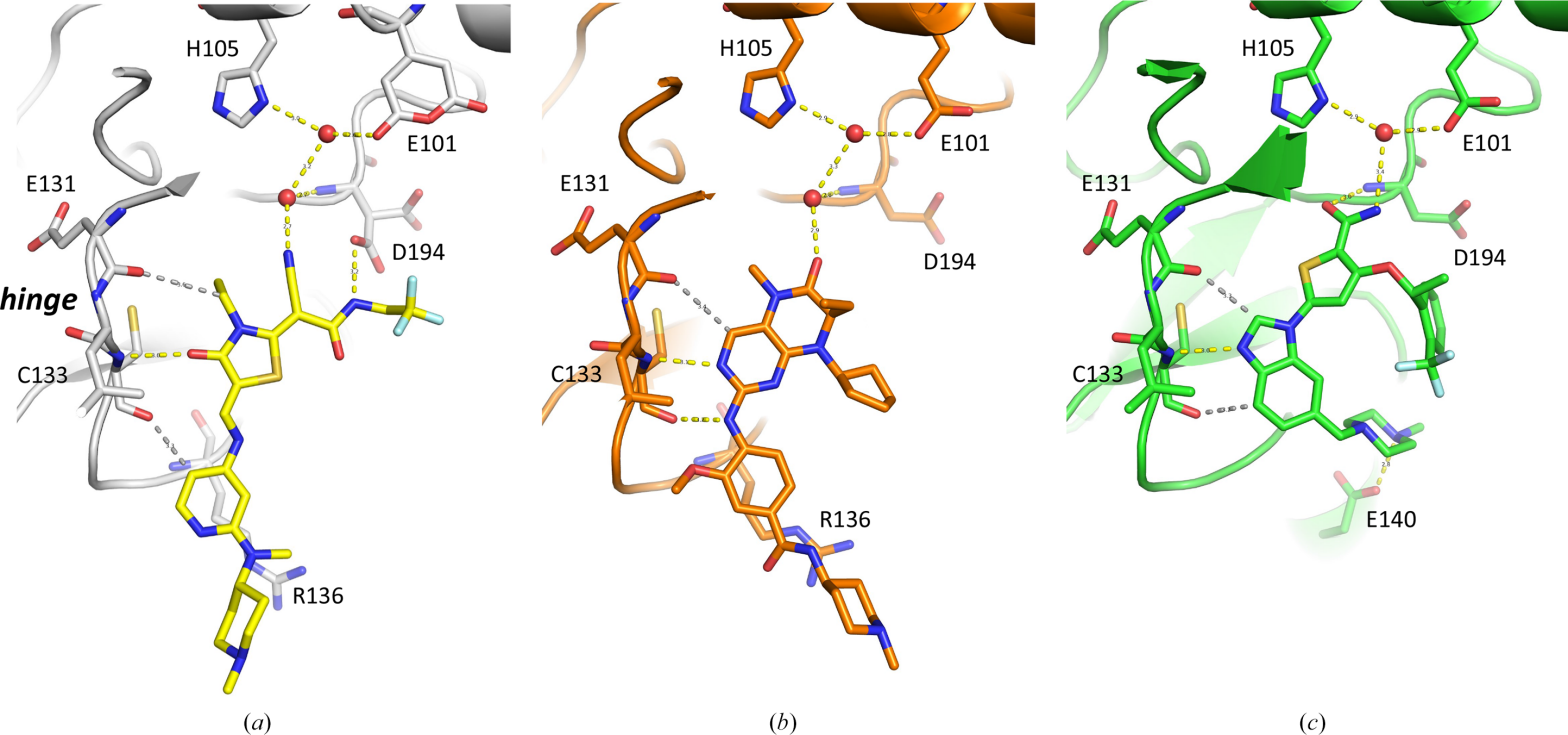
Molecular binding modes of three different PLK1 inhibitor classes. Comparison of three co-crystal structures with PLK1^K225D/K226A^ in (*a*) with compound 1 (C atoms in yellow), in (*b*) with BI 2536 (C atoms in orange) and in (*c*) with GSK461364 (C atoms in green). Classical hydrogen bonds are shown as yellow dotted lines; nonclassical (C—H⋯O=C) hydrogen bonds are depicted as grey dotted lines.

The new and robust PLK1^K225D/K226A^ crystallization system reported here enabled us to elucidate the molecular binding modes of three chemically unrelated but highly potent PLK1 inhibitor classes (Fig. 1 ▸ and Table 4 ▸). We identified the thiazolidinone inhibitor class represented by ZK-TAL (Fig. 1 ▸*a*) in an in-house high-throughput screening campaign. It potently inhibits PLK1 and triggers cell-cycle arrest (Santamaria *et al.*, 2007 ▸). The co-crystal structure reported here of the closely related compound 1 in complex with PLK1^K225D/K226A^ reveals the molecular binding mode of this inhibitor class. In parallel, we also determined the molecular binding mode of BI 2536, which was an early clinical candidate (Frost *et al.*, 2012 ▸; Mross *et al.*, 2012 ▸) and a close relative of the clinical candidate Volasertib (BI-6727; Rudolph *et al.*, 2009 ▸). The obtained structures with BI 2536, both with PLK1^K225D/K226A^ and with PLK1^WT^ in complex with the DARPin, are consistent with the binding mode in the crystal structure of PLK1^T210V^ (Kothe, Kohls, Low, Coli, Cheng *et al.*, 2007 ▸). Finally, we also report the crystal structure of PLK1 in complex with GSK461364 (Olmos *et al.*, 2011 ▸), another PLK1 inhibitor which has been in clinical trials. Fig. 8 ▸ shows a comparison of these three different inhibitor classes which all target the same ATP-binding site of PLK1.

All three chemical classes target the central hydrogen-bond donor in the hinge region, the backbone nitrogen of Cys133. Only BI 2536 can form a second classical hydrogen bond to the hinge region, by donating a 3.2 Å hydrogen bond to the backbone carbonyl O atom of Cys133 via the amino group of the aminopyrimidine scaffold. In the thiazolidinone compound 1, this second interaction point at the hinge region of PLK1 is targeted via a weak or nonclassical hydrogen bond (Weiss *et al.*, 2001 ▸) donated by a C—H group of the outer phenyl ring (3.3 Å; grey dotted line in Fig. 8 ▸*a*). In the co-crystal structure with the inhibitor GSK461364, it is addressed via a similar nonclassical hydrogen bond donated by a C—H group of the benzo moiety of the benzoimidazole scaffold (3.2 Å; Fig. 8 ▸*c*). The third hydrogen-bond anchor point available at the hinge region of PLK1, the carbonyl oxygen of Glu131, is targeted by all three inhibitor classes only via non­classical hydrogen bonds: in compound 1 via a C—H group of the ethyl moiety (3.6 Å), in BI 2536 via a C—H group of the pyrimidine ring (3.4 Å) and in GSK461364 via a C—H group of the imidazole ring (3.3 Å). The back pocket behind the ATP-binding side offers the backbone nitrogen of Asp194 of the DFG motif as a hydrogen-bond interaction partner (Fig. 8 ▸). Here, the three inhibitor classes differ: GSK461364 inserts deepest and addresses this backbone nitrogen via a direct hydrogen bond to its amide group. Compound 1 does not insert as deeply and instead forms a water-bridged hydrogen bond via its nitrile moiety. Similarly, BI 2536 does not insert as deeply either and also targets the backbone NH atom of Asp194 via a water-bridged hydrogen bond.

GSK reported that the benzimidazole inhibitor class developed for PLK1 had also been redesigned into inhibitors targeting the kinase IKKɛ, and while IKKɛ could not be crystallized at that time, Bamborough and coworkers used the kinase CDK2 as a surrogate to elucidate the most likely binding mode of this inhibitor class in IKKɛ (Bamborough *et al.*, 2006 ▸). The crystal structure of CDK2 in complex with the benzimidazole variant compound 6 from Bamborough and coworkers (PDB entry 2i40) indeed shows the same overall binding mode as now observed in our co-complex structure of GSK461364 with PLK1^K225D/K226A^, although GSK461364 is not active against CDK2 and IKKe. This indicates that identical hinge-binding scaffolds can indeed be optimized to become highly selective for very different kinase targets.

## 5. Summary and outlook

The surface modification of PLK1 identified in this study following the surface-entropy reduction (SER) approach yielded a protein which crystallizes readily and forms an excellent new crystallization platform for PLK1–inhibitor co-crystal structures. The new PLK1 co-crystal structures presented here widen the understanding of how small-molecule inhibitors can bind into the ATP-binding site of PLK1. Three different chemical classes, developed independently by three different pharmaceutical companies, adopt very different ways to interact with the ATP-binding pocket. The extended structural understanding presented in this contribution will support future modelling approaches to design inhibitors for PLK1 and for related kinases.

## Supplementary Material

PDB reference: PLK1^K225D/K226A^, complex with compound 1, 9r1w

PDB reference: complex with BI 2536, 9r1x

PDB reference: complex with GSK461364, 9r1y

PDB reference: PLK1^WT^:DARPin, complex with compound 1, 9r8b

PDB reference: complex with BI 2536, 9r8c

Chemistry, General Methods and Materials, Supplementary Figures and Table. DOI: 10.1107/S2059798325009325/chr5007sup1.pdf

## Figures and Tables

**Table 1:** PLK1 surface-entropy reduction mutants generated in this study

Construct	Mutations	Localization in kinase domain	Solubility
1	K61D/K66D	P loop	−
2	E206D/K208D/K209A	Activation segment	−
3	K225D/K226A	Adjacent to activation segment	+
4	K257D/E258D	Start of an exposed helix	+
5	K264D/K265D/E267D	End of an exposed helix	+
6	K143D/K146D	After hinge	−
7	E206D/K208D/K209A/K225D/K226A	Combination of 2 and 3	−
8	K61D/K66D/K257D/E258D	Combination of 1 and 4	−
9	K143D/K146D/K264D/K265D/E267D	Combination of 6 and 5	−

**Table 2:** Data collection and processing Values in parentheses are for the outer shell.

Data set	PLK1^K225D/K226A^:compound 1	PLK1^K225D/K226A^:BI 2536	PLK1^K225D/K226A^:GSK461364	PLK1^WT^:DARPin:compound 1	PLK1^WT^:DARPin:BI 2536
Diffraction source	BL14.2, BESSY	BL14.2, BESSY	BL14.2, BESSY	ID14-1, ESRF	ID29, ESRF
Wavelength (Å)	0.9537	0.91841	0.91841	0.934	1.037
Temperature (K)	100	100	100	100	100
Detector	MAR165 CCD	MAR165 CCD	MAR165 CCD	ADSC Quantum 4	ADSC Quantum 315
Crystal-to-detector distance (mm)	120	220	190	235.7	330.28
Rotation range per image (°)	1.0	1.0	0.5	0.2	1.0
Total rotation range (°)	180	180	213	66.8	120.0
Exposure time per image (s)	n.a	n.a.	n.a.	1.6	0.5
Space group	*C*2	*C*2	*C*2	*P*2_1_2_1_2_1_	*P*2_1_2_1_2_1_
*a*, *b*, *c* (Å)	113.1, 103.9, 70.8	112.5, 104.5, 71.3	113.8, 104.2, 71.3	61.1, 136.9, 140.7	62.6, 135.3, 136.7
α, β, γ (°)	90, 115.4, 90	90, 115.6, 90	90, 115.3, 90	90, 90, 90	90, 90, 90
Mosaicity (°)	0.56	0.65	0.96	0.12	0.30
Resolution range (Å)	36.90–2.20 (2.28–2.20)	42.18–2.84 (2.93–2.84)	42.43–2.60 (2.68–2.60)	68.54–2.20 (2.33–2.20)	48.08–2.24 (2.38–2.24)
Total No. of reflections	116574 (5102)	55805 (1488)	84640 (2831)	161236 (23995)	233157 (28100)
No. of unique reflections	32885 (1960)	16036 (777)	20523 (1048)	56436 (8825)	55406 (8235)
Completeness (%)	87.7 (52.5)	91.5 (53.8)	88.9 (54.3)	92.5 (90.7)	98.0 (91.8)
Multiplicity	3.5 (2.6)	3.5 (1.9)	4.1 (2.7)	2.9 (2.7)	4.7 (3.8)
〈*I*/σ(*I*)〉	14.5 (2.0)	10.4 (2.1)	14.9 (4.1)	13.8 (1.4)†	15.5 (1.2)†
*R*_r.i.m._	0.085 (0.424)	0.123 (0.412)	0.071 (0.231)	0.059 (0.866)	0.085 (1.214)
CC_1/2_	n.a.	n.a.	n.a.	0.999 (0.596)	0.999 (0.538)
Overall *B* value from Wilson plot (Å^2^)	32.4	33.3	45.3	43.7	50.7

† For PLK1^WT^:DARPin:compound 1 the resolution was cut at a CC_1/2_ of 60%; *I*/σ(*I*) falls below 2.0 at about 2.3 Å resolution. For PLK1^WT^:DARPin:BI 2536, the resolution was cut at a shell completeness of about 90%; *I*/σ(*I*) falls below 2.0 at about 2.3 Å resolution.

**Table 3:** Structure solution and refinement Values in parentheses are for the outer shell.

Data set	PLK1^K225D/K226A^:compound 1	PLK1^K225D/K226A^:BI 2536	PLK1^K225D/K226A^:GSK461364	PLK1^WT^:DARPin:compound 1	PLK1^WT^:DARPin:BI 2536
PDB code	9r1w	9r1x	9r1y	9r8b	9r8c
Resolution range (Å)	36.90–2.20 (2.25–2.20)	42.18–2.84 (2.92–2.84)	42.43–2.60 (2.67–2.60)	68.54–2.20 (2.20–2.25)	48.13–2.24 (2.30–2.24)
Completeness (%)	87.3	91.1	88.59	92.42	98.1
σ Cutoff	n/a	n/a	n/a	n/a	n/a
No. of reflections, working set	31239	15216	19487	53613 (3536)	52521 (3171)
No. of reflections, test set	1641	817	1031	2826 (217)	2826 (195)
Final *R*_work_	0.178 (0.260)	0.219 (0.301)	0.185 (0.235)	0.188 (0.319)	0.188 (0.340)
Final *R*_free_	0.216 (0.316)	0.278 (33.5)	0.218 (0.241)	0.239 (0.359)	0.236 (0.368)
Cruickshank DPI	0.205	0.473	0.304	0.200	0.199
No. of non-H atoms					
PLK1, chain *A*	2474	2441	2442	2402	2371
PLK1, chain *B*	2385	2353	2365	2339	2326
DARPin, chain *C*	n.a.	n.a.	n.a.	996	981
DARPin, chain *D*	n.a.	n.a.	n.a.	986	1000
Inhibitor, chain *A*	36	38	38	36	38
Inhibitor, chain *B*	36	38	38	36	38
Glycerol	30	6	30	12	24
Sulfate/chloride ion	31	35	41	1	n.a.
Water	185	64	56	388	329
Total	5177	4968	5010	7196	7107
R.m.s. deviations					
Bond lengths (Å)	0.005	0.003	0.004	0.012	0.005
Angles (°)	1.323	1.229	1.256	1.735	1.352
Average *B* factors (Å^2^)					
Overall	61.7	58.2	68.1	61.3	50.4
PLK1, chain *A*	56.9	58.0	65.0	51.9	53.7
PLK1, chain *B*	67.5	63.5	74.1	54.5	55.6
DARPin, chain *C*	n.a.	n.a.	n.a.	60.5	50.4
DARPin, chain *D*	n.a.	n.a.	n.a.	59.7	48.4
Inhibitor, chain *A*	75.0	50.1	51.5	53.3	46.3
Inhibitor, chain *B*	91.1	49.7	56.8	66.9	43.3
Glycerol	72.0	61.4	86.0	78.5	78.4
Sulfate ion/chloride ion	88.5	109.1	108.9	82.6	n.a.
Water	48.6	36.2	51.8	53.9	48.8
Ramachandran plot					
Most favoured (%)	96.7	94.6	95.0	97.5	96.4
Allowed (%)	100.0	100.0	99.5	100.0	99.9

**Table 4:** Inhibitory activities of PLK1 inhibitors investigated in this study IC_50_ values from a high-ATP (10 m*M*) *in vitro* PLK1 kinase-activity assay.

Compound	IC_50_ (n*M*)
TAL	177.5 ± 52.6 (*n* = 3)
Compound 1	3.6 ± 2.3 (*n* = 4)
BI 2536	5.6 ± 0.7 (*n* = 4)
GSK461364	2.5 (*n* = 1)
