# Supplementary material for: Polo-like kinase 1–inhibitor co-complex structures via the surface-entropy reduction approach and a DARPin-assisted approach
Source: Acta Crystallogr D Struct Biol. 2025 Nov 17;81(Pt 12):718–33. doi: 10.1107/S2059798325009325 (PMC12809504; doi:10.1107/S2059798325009325)
Supplement: Supplementary file 1 [file d-81-00718-sup1.pdf]

STRUCTURAL BIOLOGY

Volume 81 (2025)

Supporting information for article:

PLK1–inhibitor co-complex structures via the surface-entropy reduction approach and a DARPin-assisted approach

Uwe Eberspaecher, Arndt A. Schmitz, Gerhard Siemeister, Ulf Bömer, Tiago M. Bandeiras, Pedro M. Matias, Volker K. Schulze and Roman C. Hillig

S1. Chemistry, General Methods and Materials

All reagents and solvents were used as purchased, unless otherwise specified. All final products were at least 95% pure, as determined by UPLC or alternatively by ^{1}H NMR. ^{1}H NMR spectra were recorded on Bruker Avance III HD spectrometers operating at 300, 400, or 500 MHz. Chemical shifts (δ) are reported in parts per million (ppm) and coupling constants (J) are given in hertz (Hz). Spin multiplicities are reported as s = singlet, d = doublet, t = triplet, q = quartet, quin = quintet, spt = septet, m = multiplet, and br = broad. HRMS were recorded on a Waters XEVO G2XS with electrospray ionization, coupled to an LC Waters Acquity i-class instrument.

S1.1. LC-MS Method 1

System: Waters Acquity UPLC-MS single quad; column: Acquity UPLC BEH C18 1.7 µm, 50 × 2.1 mm; solvent A: water + 0.2 vol % aqueous ammonia (32%), solvent B: acetonitrile; gradient: 0–1.6 min 1–99% B, 1.6–2.0 min 99% B; flow: 0.8 mL/min; temperature: 333 K; DAD scan: 210–400 nm.

S1.2. Synthesis of compound 1

Compound 1 ((2Z)-2-cyano-2-{3-ethyl-5-[({2-[methyl(1-methylpiperidin-4-yl)amino]pyridin-4-yl}amino)methylidene]-4-oxo-1,3-thiazolidin-2-ylidene}-N-(2,2,2-trifluoroethyl)acetamide) was synthesized in 7 steps as outlined in Suppl. Fig. 1 (Scheme 1).

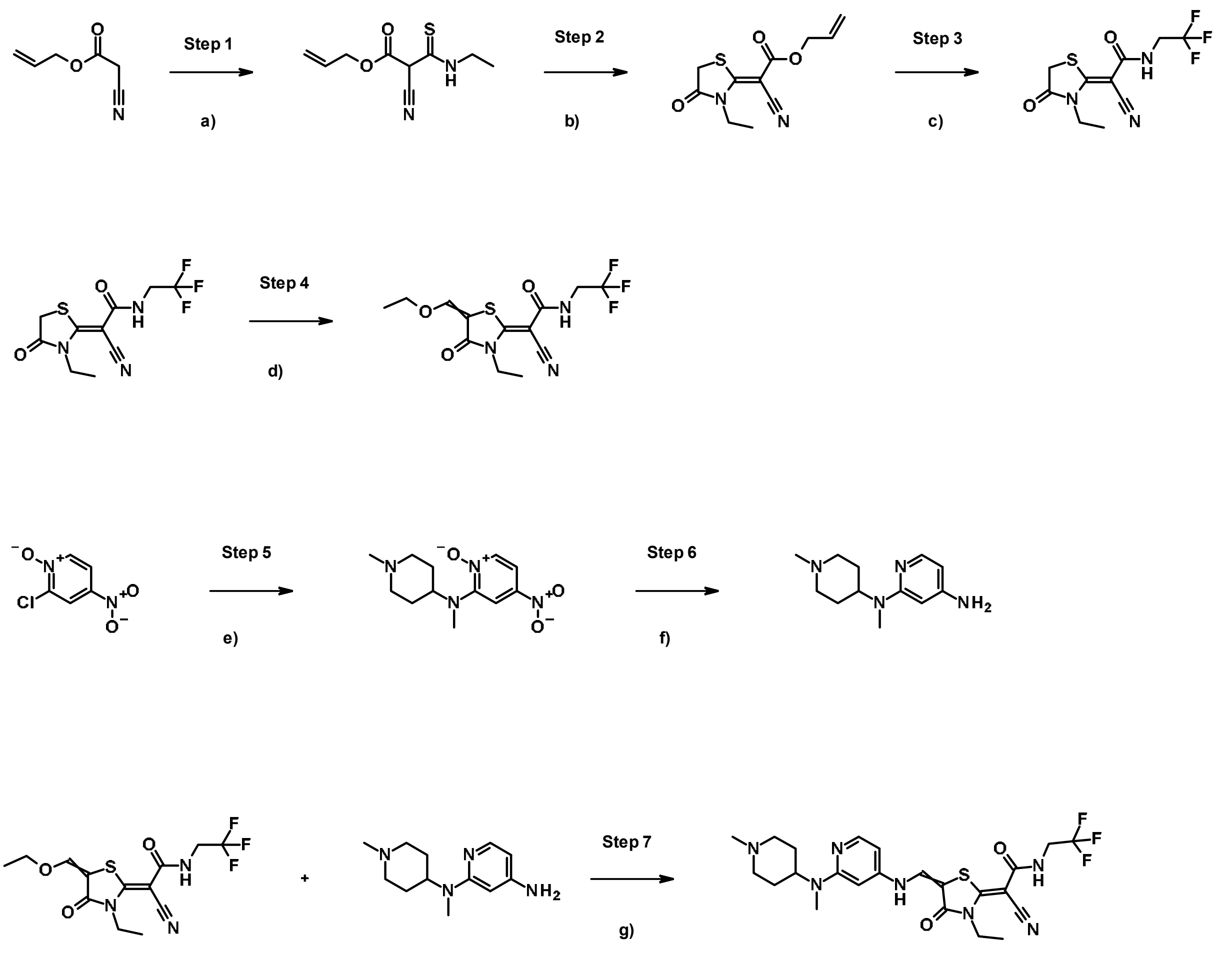

Compound 1

Figure S1 Scheme 1: Hünig base, EtNCS, 333 K, 8 h, 313 K, 40 h; b) bromoacetyl chloride, THF, r.t., 16 h, 54% yield for 2 steps; c) i) 1,3-dimethyl-barbituric acid, $Pd(PPh_3)_4$, r.t., 2 h, ii) TBTU, Hünig base, 2,2,2-trifluoroethanamine, r.t., 16 h, 79% yield; d) $(EtO)_3CH$, Ac_2O, 423 K, 20 h; 84% yield; e) N,1-dimethylpiperidin-4-amine, Hünig base, 1-propanol, 3737 K, 2 h, 32% yield; f) ammonium formate, Pd/C, ethanol, reflux, 18 h, 99% yield; g) reflux, 2 h, 73% yield.

S1.2.1. Synthesis Step 1: Prop-2-en-1-yl 2-cyano-3-(ethylamino)-3-sulfanylidenepropanoate

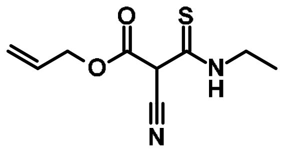

To a stirred solution of prop-2-en-1-yl cyanoacetate (22.4 g, 179 mmol) was slowly added N,N-diisopropylethylamine (30 ml) and then isothiocyanatoethane (19 ml, 220 mmol). The mixture was stirred at 333 K for 8 h and then at room temperature for 40 h. Water was added, and then the mixture was acidified with hydrochloric acid and the mixture was extracted with ethyl acetate. The organic

phase was washed with saturated sodium chloride solution, dried (sodium sulfate), filtered and the solvent was removed in vacuum to give 37.4 g (98% yield) of the title compound as a crude product that was used without further purification for the next step.

S1.2.2. Synthesis Step 2: Prop-2-en-1-yl (2Z)-cyano(3-ethyl-4-oxo-1,3-thiazolidin-2-ylidene)acetate

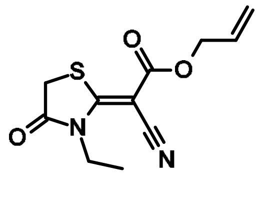

To a stirred solution of prop-2-en-1-yl 2-cyano-3-(ethylamino)-3-sulfanylidenepropanoate (37.4 g, 176 mmol) in THF (750 mL) was added bromoacetyl chloride (17 mL, 210 mmol) and the mixture was stirred at room temperature for 16 h. An aqueous solution of sodium bicarbonate was added, and the mixture was extracted with ethyl acetate. The organic phase was washed with saturated sodium chloride solution, dried (sodium sulfate), filtered and the solvent was removed in vacuum. Crystallization from ethanol gave 21.3 g (48% yield) of the title compound. The mother liquid was concentrated, and silica gel chromatography gave a solid that was crystallized from ethanol to give further 3.4 g (7.6% yield) of the title compound.

^{1}H NMR (300 MHz, DMSO) δ [ppm] = 5.93 (tdd, *J* = 5.18, 10.46, 17.24 Hz, 1H), 5.16-5.40 (m, 2H), 4.67 (td, *J* = 1.58, 5.13 Hz, 2H), 4.08 (q, *J* = 7.16 Hz, 2H), 3.92 (s, 2H), 1.14-1.22 (m, 3H).

S1.2.3. Synthesis Step 2: (2Z)-2-cyano-2-(3-ethyl-4-oxo-1,3-thiazolidin-2-ylidene)-N-(2,2,2-trifluoroethyl)acetamide

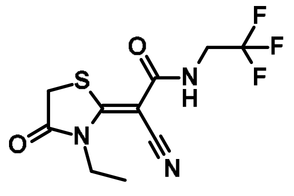

To a stirred solution of prop-2-en-1-yl (2Z)-cyano(3-ethyl-4-oxo-1,3-thiazolidin-2-ylidene)acetate (10.2 g, 40.4 mmol) and 1,3-dimethylpyrimidine-2,4,6(1H,3H,5H)-trione (12.6 g, 80.9 mmol) in THF (760 mL) was added tetrakis(triphenylphosphin)palladium (934 mg, 809 µmol) and the mixture was stirred at room temperature for 2 h. Then, N,N-diisopropylethylamine (35 ml, 200 mmol), 2,2,2-trifluoroethanamine (9.5 ml, 120 mmol) and O-(Benzotriazol-1-yl)-N,N,N',N'-tetramethyluronium-tetrafluoroborat (38.9 g, 121 mmol) were added and the mixture was stirred at room temperature for

16 h. Water was added, the mixture was stirred for 5 min and the mixture was extracted with ethyl acetate. The organic phase was washed with half-saturated sodium chloride solution, dried (sodium sulfate), filtered and the solvent was removed in vacuum. Crystallization from ethanol gave 9.31 g (78.5% yield) of the title compound

^{1}H NMR (400 MHz, DMSO) δ [ppm] = 8.30 (t, *J* = 6.32 Hz, 1H), 4.07 (q, *J* = 7.07 Hz, 2H), 3.90 (dq, *J* = 6.44, 9.56 Hz, 2H), 3.81 (s, 2H), 1.18 (t, *J* = 7.07 Hz, 3H).

S1.2.4. Synthesis Step 3: (2Z)-2-cyano-2-[5-(ethoxymethylidene)-3-ethyl-4-oxo-1,3-thiazolidin-2-ylidene]-N-(2,2,2-trifluoroethyl)acetamide

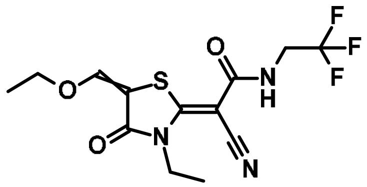

To a stirred solution of (2Z)-2-cyano-2-(3-ethyl-4-oxo-1,3-thiazolidin-2-ylidene)-N-(2,2,2-trifluoroethyl)acetamide (7.10 g, 24.2 mmol) and (diethoxymethoxy)ethane (97 ml, 580 mmol; CAS-RN: 122-51-0) was added acetic anhydride (21 ml, 220 mmol) and the mixture was stirred at 423 K for 20 h. The mixture was allowed to cool to room temperature; ethanol was added and a solid precipitated. The precipitate was collected by filtration, washed with ethanol and hexanes and dried in vacuum to give 7.07 g (83.6% yield) of the title compound.

^{1}H NMR (300 MHz, DMSO) δ [ppm] = 8.35 (t, *J* = 6.31 Hz, 1H), 8.05 (s, 1H), 4.35 (q, *J* = 7.16 Hz, 2H), 4.20 (q, *J* = 7.03 Hz, 2H), 3.95 (dq, *J* = 6.41, 9.54 Hz, 2H), 1.30 (t, *J* = 7.16 Hz, 3H), 1.24 (t, *J* = 7.06 Hz, 3H).

S1.2.5. Synthesis Step 4: N-methyl-N-(1-methylpiperidin-4-yl)-4-nitropyridin-2-amine 1-oxide

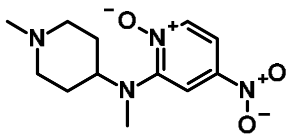

To a stirred solution of 2-chloro-4-nitropyridine 1-oxide (4.00 g, 22.9 mmol) in 1-propanol (40 mL) was added N,N-diisopropylethylamine (7.8 ml, 46 mmol), and N,1-dimethylpiperidin-4-amine (3.7 ml, 25 mmol). The mixture was stirred at 373 K. for 2 h. The solvent was removed in vacuum, and the crude product was purified by aminophase-silica gel chromatography followed by silica gel chromatography to give a residue that was dissolved in a mixture of ethyl acetate and methanol (10:1). The organic phase was washed with half-saturated sodium chloride solution (three times), dried (sodium sulfate), filtered and the solvent was removed in vacuum to give 1.95 g (32% yield) of the title compound.

^{1}H NMR (300 MHz, DMSO) δ [ppm] = 8.13-8.39 (m, 1H), 7.63-7.82 (m, 2H), 3.78-3.94 (m, 1H), 2.84 (s, 3H), 2.77-2.83 (m, 2H), 2.14 (s, 3H), 1.70-1.96 (m, 4H), 1.52-1.66 (m, 2H).

S1.2.6. Synthesis Step 5: N2-methyl-N2-(1-methylpiperidin-4-yl)pyridine-2,4-diamine

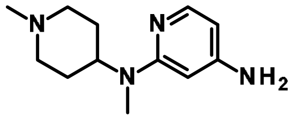

To a stirred solution of N-methyl-N-(1-methylpiperidin-4-yl)-4-nitropyridin-2-amine 1-oxide (925 mg, 3.47 mmol) in ethanol (61 ml) was added ammonium formate (3.29 g, 52.1 mmol) and palladium on carbon (10% w/w palladium; 37.0 mg, 34.7 µmol) and the mixture was stirred at reflux for 18 h. The mixture was filtered, washed with a mixture of dichloromethane and methanol (2:1; 200 mL) and the solution was concentrated in vacuum. Aminophase-silica gel chromatography gave 760 mg (99% yield) of the title compound.

^{1}H NMR (400 MHz, DMSO) δ [ppm] = 7.54 (d, *J* = 5.56 Hz, 1H), 5.79 (dd, *J* = 1.77, 5.56 Hz, 1H), 5.63 (d, *J* = 1.52 Hz, 1H), 5.52 (s, 2H), 4.33 (tt, *J* = 4.04, 11.87 Hz, 1H), 2.72-2.84 (m, 2H), 2.65 (s, 3H), 2.12 (s, 3H), 1.89 (dt, *J* = 2.27, 11.75 Hz, 2H), 1.65 (dq, *J* = 3.66, 12.17 Hz, 2H), 1.35-1.46 (m, 2H).

S1.2.7. Synthesis Step 7: Compound 1, (2Z)-2-cyano-2-{3-ethyl-5-[({2-[methyl(1-methylpiperidin-4-yl)amino]pyridin-4-yl}amino)methylidene]-4-oxo-1,3-thiazolidin-2-ylidene}-N-(2,2,2-trifluoroethyl)acetamide

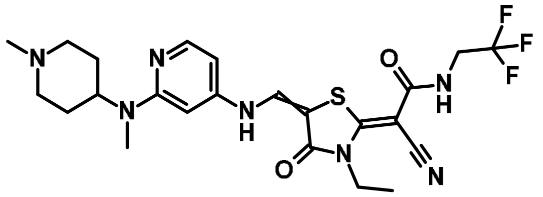

To a stirred solution of N^2-methyl-N^2-(1-methylpiperidin-4-yl)pyridine-2,4-diamine (135 mg, 613 µmol) in ethanol (9.0 ml) was added (2Z)-2-cyano-2-[5-(ethoxymethylidene)-3-ethyl-4-oxo-1,3-thiazolidin-2-ylidene]-N-(2,2,2-trifluoroethyl)acetamide (214 mg, 613 µmol), and the mixture was stirred at reflux for 2 h. The solvent was removed in vacuum, and the crude product was purified by aminophase-silica gel chromatography followed by silica gel chromatography to give a solid that was triturated dichloromethane to give 235 mg (73% yield) of the title compound.

LC-MS (Method 1): ^{t}R = 1.09 min. MS (ESI-): *m*/*z* = 522 [M - H]$^-$.

HRMS (ESI): *m*/*z* calcd for $C_{23}H_{28}F_3N_7O_2S$ [M + H]$^+$: 524.2056; found: 524.2050.

^{1}H NMR (300 MHz, DMSO-d_6) δ [ppm] = 10.07-10.43 (m, 1H), 8.27 (t, *J* = 6.31 Hz, 1H), 8.18 (s, 1H), 7.95 (d, *J* = 5.65 Hz, 1H), 6.55 (dd, *J* = 1.60, 5.75 Hz, 1H), 6.39 (d, *J* = 1.32 Hz, 1H), 4.37-4.53 (m, 1H), 4.17-4.32 (m, 2H), 3.88-4.06 (m, 2H), 2.87 (br d, *J* = 10.55 Hz, 2H), 2.82 (s, 3H), 2.21 (s, 3H), 1.94-2.11 (m, 2H), 1.67-1.86 (m, 2H), 1.51 (br d, *J* = 9.80 Hz, 2H), 1.26 (t, *J* = 6.97 Hz, 3H).

^{13}C NMR (151 MHz, DMSO-d_6) δ [ppm] = 166.22, 165.16, 163.01, 159.51, 148.82, 148.44, 135.40, 124.76 (q, *J* = 279.3 Hz, 1C), 117.18, 99.70, 94.75, 92.10, 71.56, 55.07 (2C), 51.47, 45.81, 40.52 (q, *J* = 33.9 Hz, 1C), 40.08, 29.64, 28.40 (2C), 14.29.

^{19}F NMR (377 MHz, DMSO-d_6) δ [ppm] = -70.71 (t, *J* = 9.73 Hz, 3F).

S2. Crystals of the SER mutant PLK1$^{K225D/K226A}$

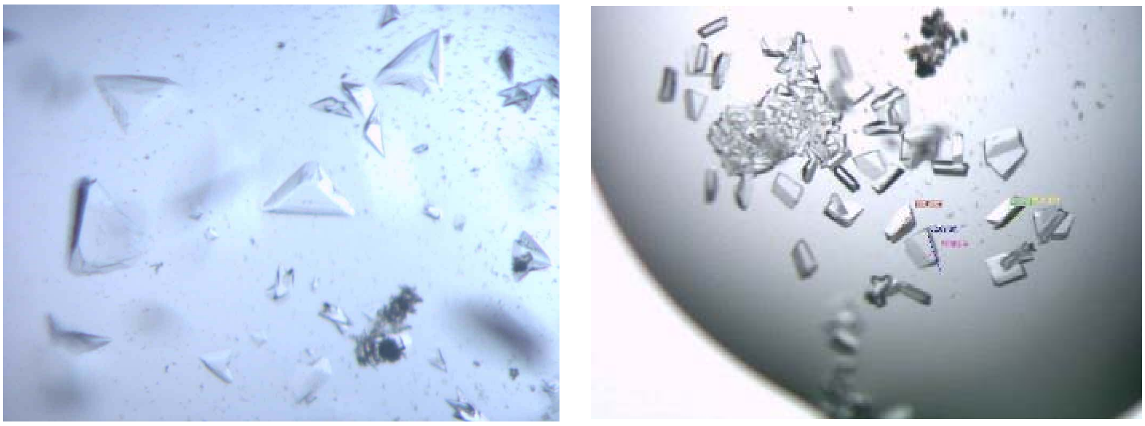

Figure S2 Crystals of the SER mutant PLK1$^{K225D/K226A}$. Shown are representative arrow-head shaped crystals of PLK1$^{K225D/K226A}$, here obtained from co-crystallization with GSK461364, in two different drops. Crystals grew to a maximum size of approx. 50 x 30 x 20 µm.

Table S1 Interactions between PLK1^{WT} and the DARPin (from complex with compound 1).

PLK1 Residue	DARPin residue	Type of interaction
Arg A134	Thr-D112	weak VdWaals contact
Arg A135	Asp-D110	**salt bridge**
Arg A136	Val D78 (C=O)	**polar interaction**
Lys A143		no direct interaction, but masked (not unfavorable anymore for crystal contact)
Arg A144	Asp D77	**salt bridge**
	Thr D46 OH	**polar interaction**
	Thr A320 OH	**polar interaction** (intramolecular)
	Leu D48	VdWaals contact
Arg A57	Met D111	weak VdWaals contact
Arg A313	Glu D123	**bidental salt bridge**
Glu A140	Val D78	VdWaals contact, forces Glu A140 to fold back and form two additionally intramolecular interactions:
	Ser A137 (OH)	**polar interaction** (intramolecular)
	Ser A137 (NH)	**polar interaction** (intramolecular)
Glu A186	Thr112	VdWaals contact
Pro A315	Glu D123	VdWaals contact
Ile A316	Phe D81 /	VdWaals contact
	Glu D123	VdWaals contact
Thr A317	Met D89	VdWaals contact
Leu A319	Tyr D79	VdWaals contact
Ile A320	Val D56	VdWaals contact
Thr A321	Val D56	VdWaals contact
